# Crystal structures and supra­molecular inter­actions of prism[6]arene-based host–guest systems with dihalohexa­nes

**DOI:** 10.1107/S2056989025003767

**Published:** 2025-05-02

**Authors:** Mickey Vinodh, Nour O. Abdeljaber, Fatemeh H. Alipour, Talal F. Al-Azemi

**Affiliations:** a Department of Chemistry, Kuwait University, PO Box 5969, Safat 13060, Kuwait; Indian Institute of Science Education and Research Bhopal, India

**Keywords:** crystal structure, pereth­oxy prism[6]arene, 1,6-di­chloro­hexa­ne, 1,6-di­iodo­hexa­ne, host–guest systems.

## Abstract

The crystal structures of the supra­molecular systems formed by pereth­oxy prism[6]arene (**PS6**) with 1,6-di­chloro­hexane (**HexCl2**), C_90_H_96_O_12_·2C_6_H_12_Cl_2_, and 1,6-di­iodo­hexane (**HexI2**), C_90_H_96_O_12_·2C_6_H_12_I_2_, are reported. **PS6** encapsulates these linear dihaloalkanes, creating 1:1 host–guest systems (**PS6·HexCl2** and **PS6·HexI2**). The structural details and host–guest inter­molecular inter­actions within the crystal networks are presented and discussed. In both crystal structures, the prism[6]arene macrocycle adopts a distorted cuboid shape, with the guest positioned perpendicular to its main axis.

## Abstract 2

An investigation is reported of the crystal structures, Hirshfeld surface analysis and supra­molecular inter­actions of pereth­oxy prism[6]arene:di­chloro­hexane and pereth­oxy prism[6]arene:di­iodo­hexane host–guest systems.

## 1. Chemical context

Prism[*n*]arenes are a class of naphthalene-based macrocyclic systems that have been reported recently (Della Sala *et al.*, 2020 ▸, 2021 ▸). They are structurally analogous to pillar[*n*]arenes, with the key difference being that the di­alk­oxy­benzene units are replaced by dialk­oxy naphthalenes. The five di­meth­oxy­naphthalene units, linked *via* methyl­ene bridges, adopt a prism-like shape and are thus named permeth­oxy prism[5]arene. Similarly, the six-membered di­eth­oxy­naphthalene units adopt a folded cuboid-shaped conformation and are referred to as pereth­oxy prism[6]arene.

Currently, the prism[*n*]arene family remains relatively small, consisting of only two known members: prism[5]arene and prism[6]arene. Suitably designed prism[*n*]arene macrocycles are expected to possess light-sensitizing properties, making them promising candidates for detection and sensing applications, as naphthalene units exhibit remarkable fluorescence (Yao & Jiang, 2020 ▸). Additionally, the large biphenyl π-system of the naphthalene moieties provides a deeper and wider cavity in macrocyclic prism[*n*]arene derivatives, enhancing their host–guest inter­actions. Both prism[5]arene and prism[6]arene exhibit a significant degree of conformational flexibility, enabling them to accommodate various guest mol­ecules within their cavities (Yang & Jiang, 2020 ▸; Liang *et al.*, 2022 ▸; Regno *et al.*, 2022 ▸, 2024 ▸; Zhang *et al.*, 2024 ▸). However, detailed structural investigations of these prism[*n*]arene systems and their host–guest inter­actions remain underdeveloped.

Recently, the present authors provided a detailed account of the guest encapsulation characteristics of permeth­oxy prism[5]arene and pereth­oxy prism[6]arene with different α,ω-di­bromo­alkanes (Vinodh *et al.*, 2025 ▸). In the present work, we discuss the crystal structures of pereth­oxy prism[6]arene (**PS6**) co-crystallized with either 1,6-di­chloro­hexane (**HexCl2**) or 1,6-di­iodo­hexane (**HexI2**). The results show that **PS6** encapsulates these linear dihaloalkanes, forming 1:1 host–guest complexes (**PS6·HexCl2** and **PS6·HexI2**). The structural details and host–guest inter­molecular inter­actions within the crystal network are presented and discussed.
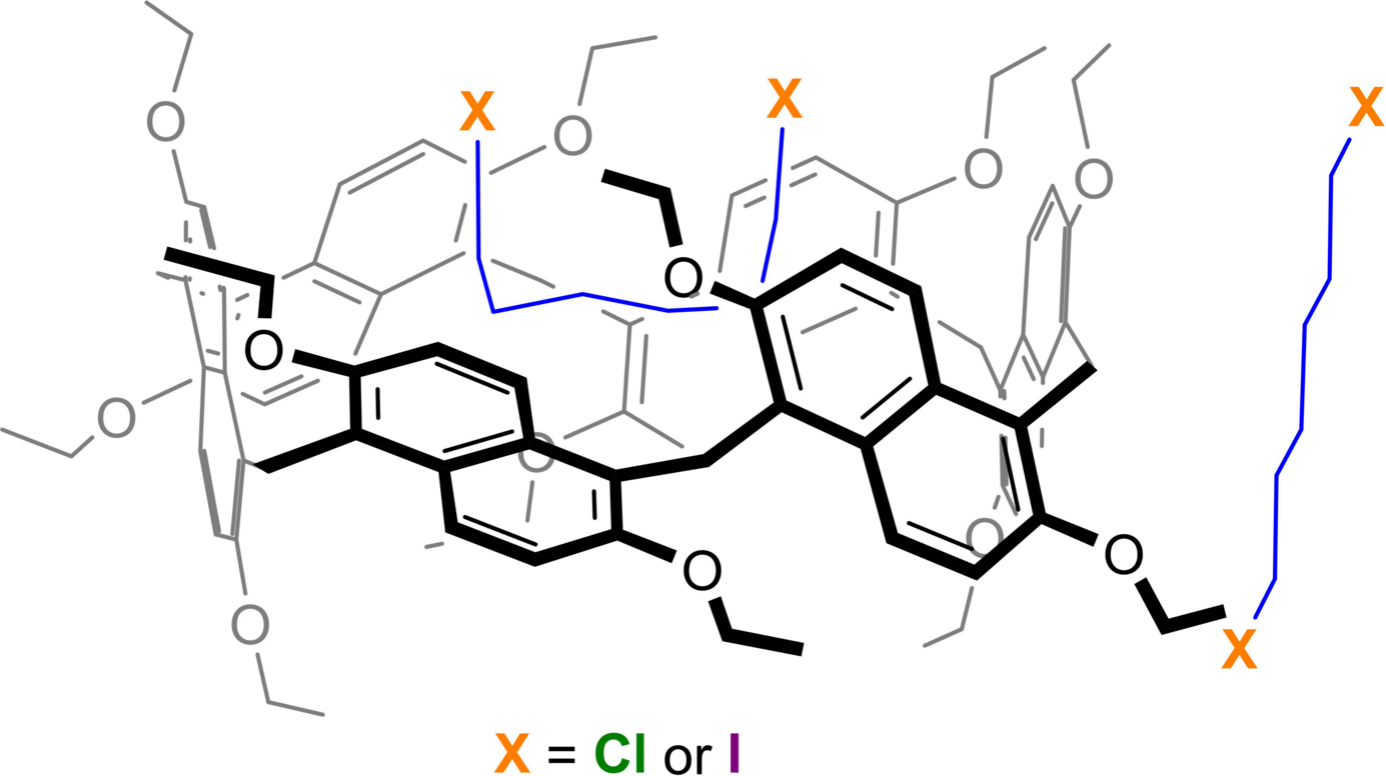


## 2. Structural commentary

**Figure 1:**
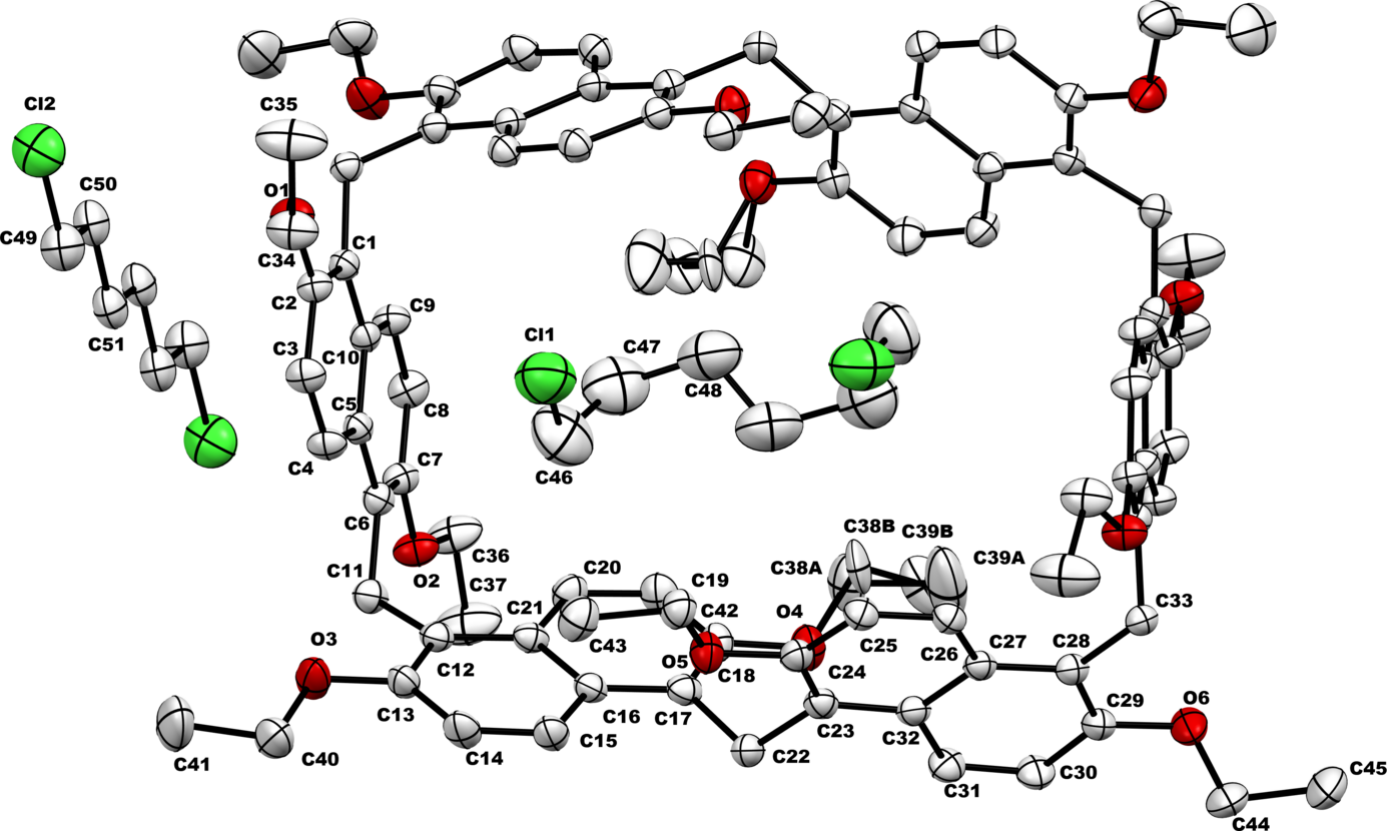
Symmetry-expanded crystal structure of **PS6·HexCl2** with displacement ellipsoids (30% probability; only the symmetry independent atoms are labeled). Hydrogen atoms are omitted for clarity.

**PS6·HexCl2** crystallizes in the monoclinic crystal system, space group *C*2*/c*, with its asymmetric unit consisting of half of a prism[6]arene mol­ecule and two halves of 1,6-di­chloro­hexane moieties. Upon symmetry expansion, one mol­ecule of 1,6-di­chloro­hexane is encapsulated within the cavity of the pereth­oxy prism[6]arene, resulting in the formation of a host–guest supra­molecular inclusion complex, **PS6·HexCl2**. Additionally, an extra 1,6-di­chloro­hexane mol­ecule is present for each **PS6·HexCl2**, acting as a space-filling solvent mol­ecule (Fig. 1 ▸). Furthermore, two disordered ethoxy groups are observed at the rim of the macrocycle in Fig. 1 ▸.

The prism[6]arene macrocycle in **PS6·HexCl2** exhibits a distorted cuboid shape, with its longer sides comprising two naphthalene units each, while the shorter sides consist of a single naphthalene unit. Measuring the distances based on the centroids of the phenyl rings in the naphthalene units, the length of the cuboid is approximately 12.32 Å, and the shortest width is 8.46 Å. Inside this cuboid-shaped prism[6]arene, a single 1,6-di­chloro­hexane guest is positioned perpendicular to the main axis of the macrocycle. This orthogonal orientation is quite different from the common threaded encapsulation typically observed in pillar[5]arenes or prism[5]arenes with such linear guest mol­ecules. As a result of this unique arrangement, the chlorine atoms of the encapsulated 1,6-di­chloro­hexane mol­ecule are aligned in the same direction and project toward the opening of the cuboid. The distance between the terminal chlorine atoms in the encapsulated guest mol­ecule is measured to be 4.83 Å, whereas in the similar 1,6-di­chloro­hexane mol­ecules positioned outside the prism[6]arene as space-filling solvents, the distance is 9.34 Å.

**Figure 2:**
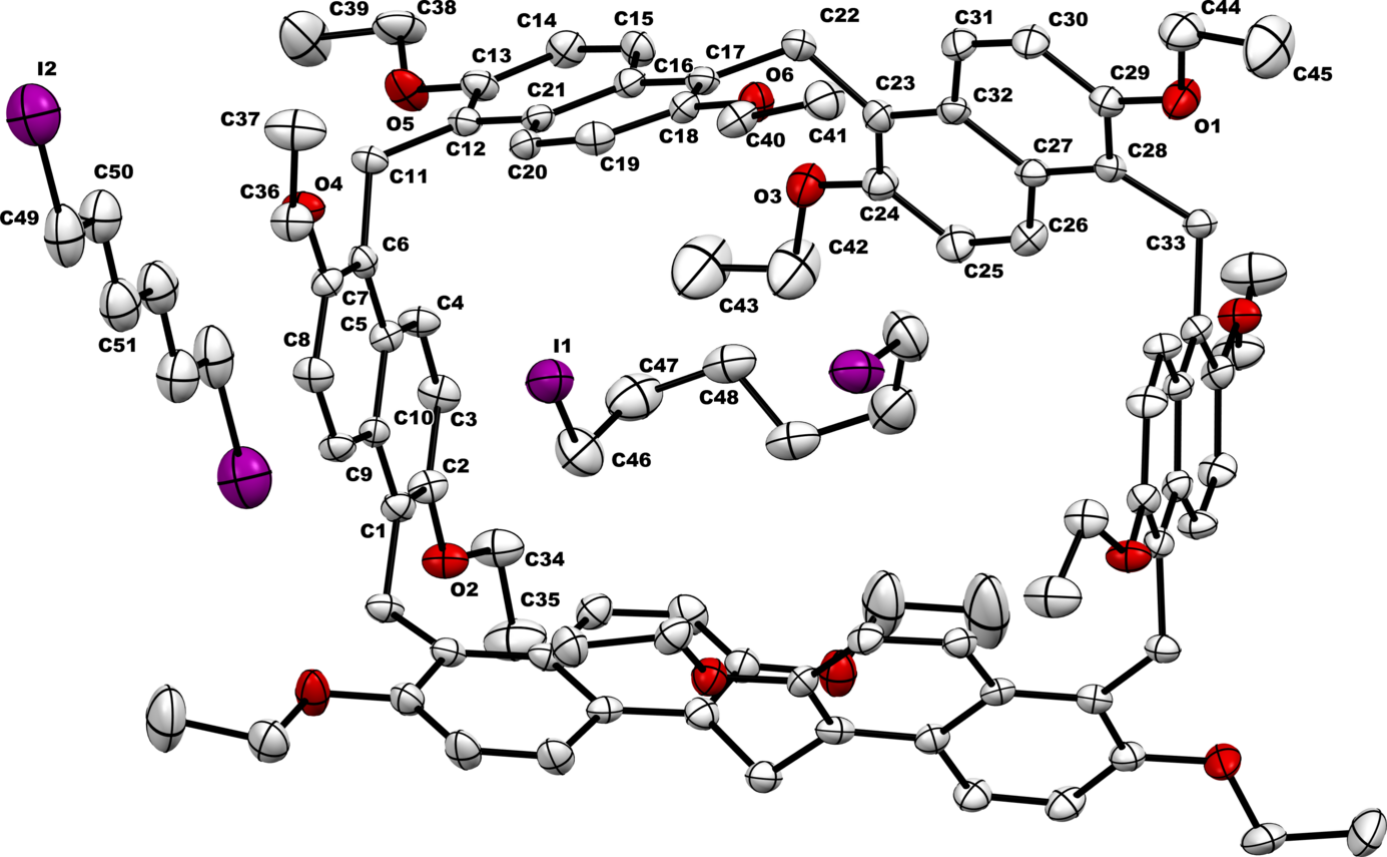
Symmetry-expanded crystal structure of **PS6·HexI2** with displacement ellipsoids (30% probability; only the symmetry independent atoms are labeled). Hydrogen atoms are omitted for clarity.

Similarly, **PS6·HexI2** crystallizes in the monoclinic crystal system, space group *C*2*/c*, with its asymmetric unit consisting of half of a prism[6]arene mol­ecule and two halves of 1,6-di­iodo­hexane moieties. Similar to **PS6·HexCl2**, the crystal structure of **PS6·HexI2** includes one mol­ecule of 1,6-di­iodo­hexane encapsulated within the cavity of pereth­oxy prism[6]arene, forming a host-guest supra­molecular inclusion complex, **PS6·HexI2**, along with an additional 1,6-di­iodo­hexane mol­ecule serving as a space-filling solvent (Fig. 2 ▸).

The prism[6]arene macrocycle in **PS6·HexI2** also exhibits a distorted cuboid shape, with a length of approximately 12.24 Å and a shortest width of around 8.23 Å. In this inclusion complex, the encapsulated 1,6-di­iodo­hexane guest is also positioned perpendicular to the main axis of the macrocycle, with its iodine atoms aligned in the same direction and projecting toward the opening of the cuboid. The distance between the terminal iodine atoms in the encapsulated guest mol­ecule is measured to be 4.56 Å, whereas in the similar 1,6-di­iodo­hexane mol­ecules positioned outside the prism[6]arene as space-filling solvents, this distance is 9.98 Å. This suggests that the 1,6-di­iodo­hexane guest experiences greater strain within the prism[6]arene cavity compared to the 1,6-di­chloro­hexane guest.

## 3. Supra­molecular features

**Figure 3:**
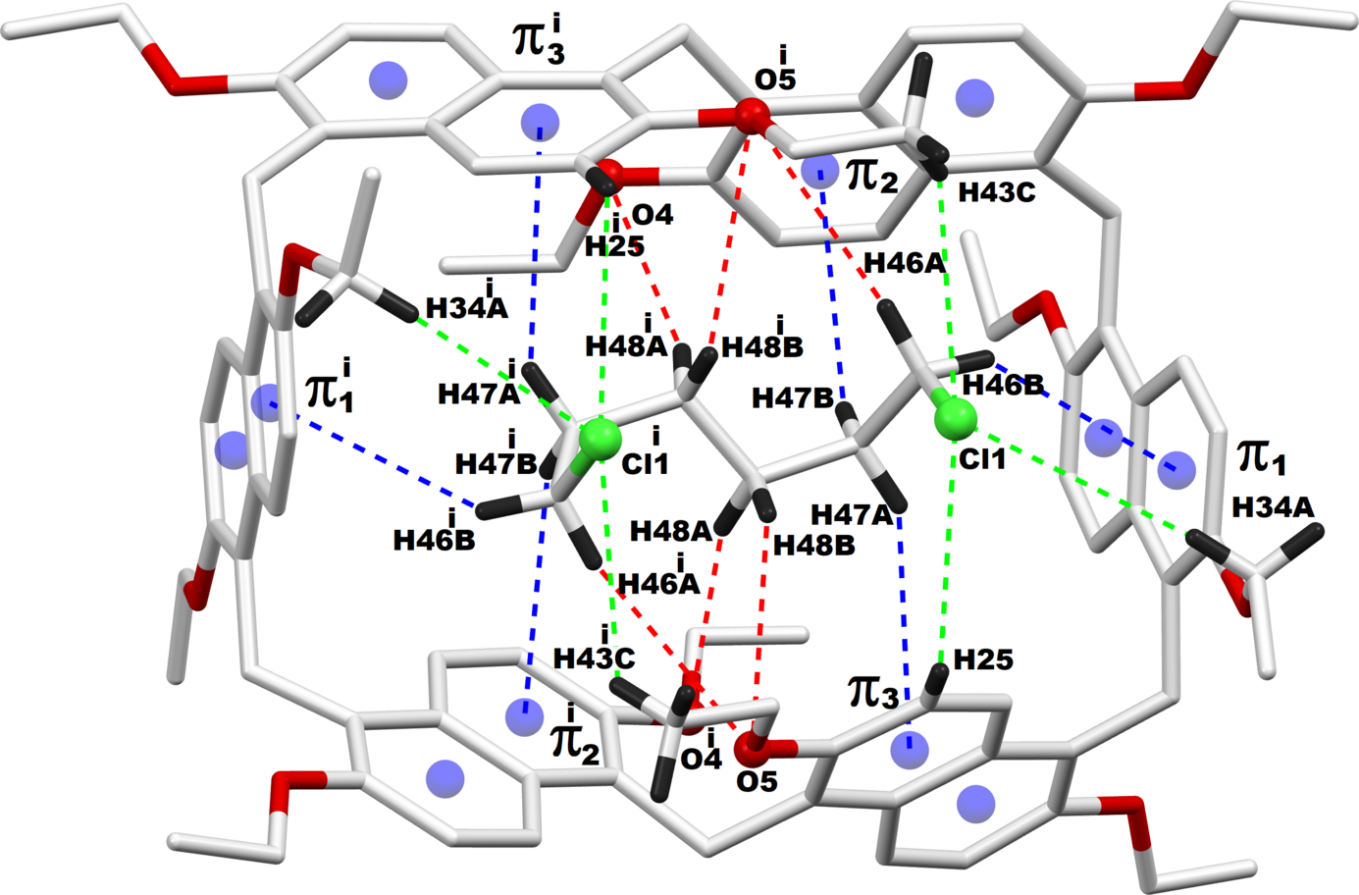
Host–guest inter­actions between the prism[6]arene host and di­chloro­hexane guest. π1–π3 are the centroids of the phenyl rings C1—C5,C10; C16—C21 and C23—C27,C32 respectively. Symmetry code (^1^) 1 − *x*, *y*, 0.5 − *z*.

**Figure 4:**
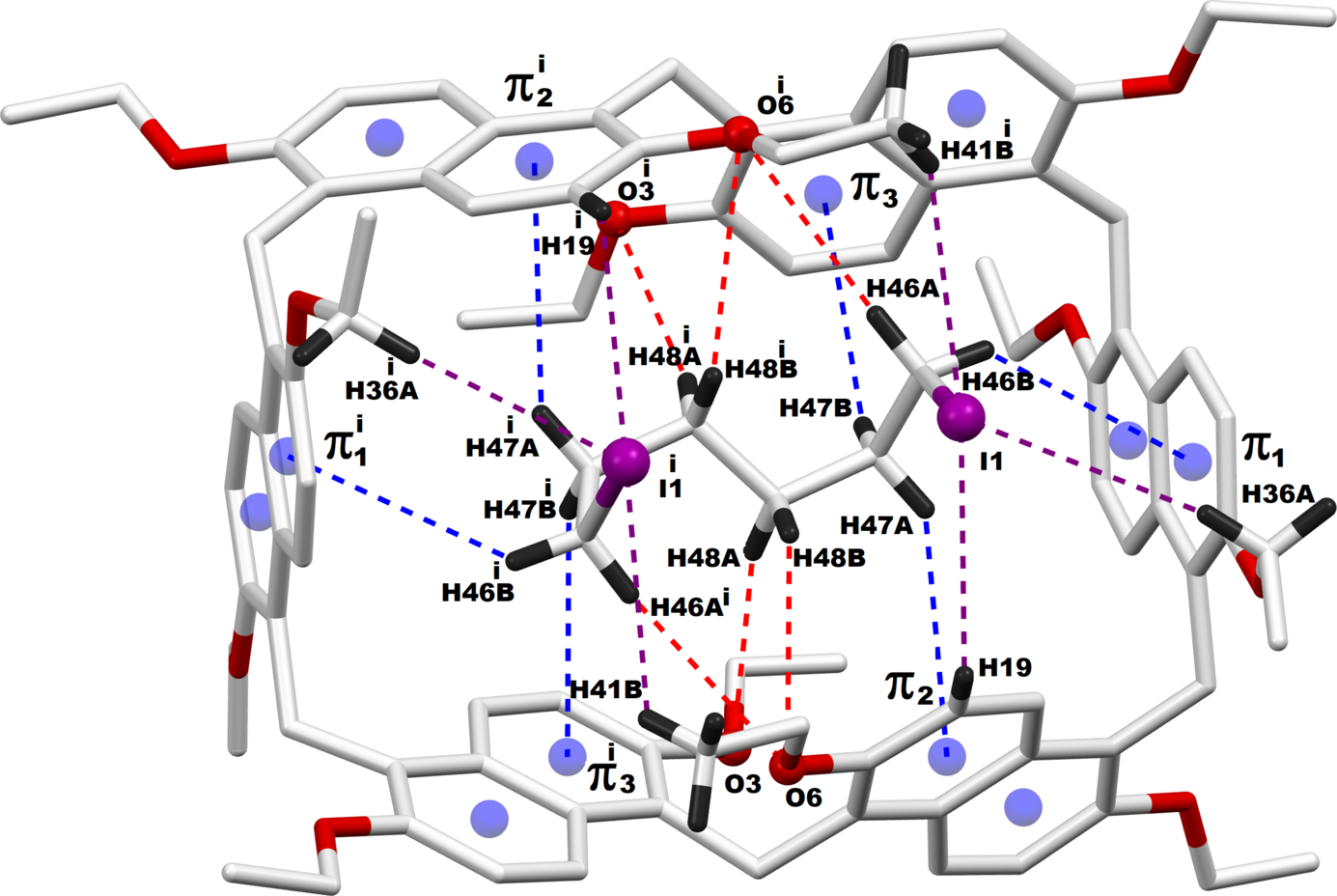
Host–guest inter­actions between the prism[6]arene host and di­iodo­hexane guest. π1–π3 are the centroids of the phenyl rings C5–C10, C16–C21 and C23–C27,C32, respectively. Symmetry code: (1) 1 − *x*, *y*, 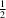 − *z*.

The encapsulated guest mol­ecules, 1,6-di­chloro­hexane or 1,6-di­iodo­hexane, engage in multiple non-bonding inter­actions with their prism[6]arene macrocyclic host *via* C—H⋯O or C—H⋯π inter­actions. Fig. 3 ▸ illustrates the host–guest inter­actions between prism[6]arene and 1,6-di­chloro­hexane in **PS6·HexCl2**, while Fig. 4 ▸ depicts the inter­actions between prism[6]arene and 1,6-di­iodo­hexane in **PS6·HexI2**. The qu­anti­tative details of these supra­molecular host–guest inter­actions in **PS6·HexCl2** and **PS6·HexI2** are presented in Tables 1 ▸ and 2 ▸, respectively.

**Figure 5:**
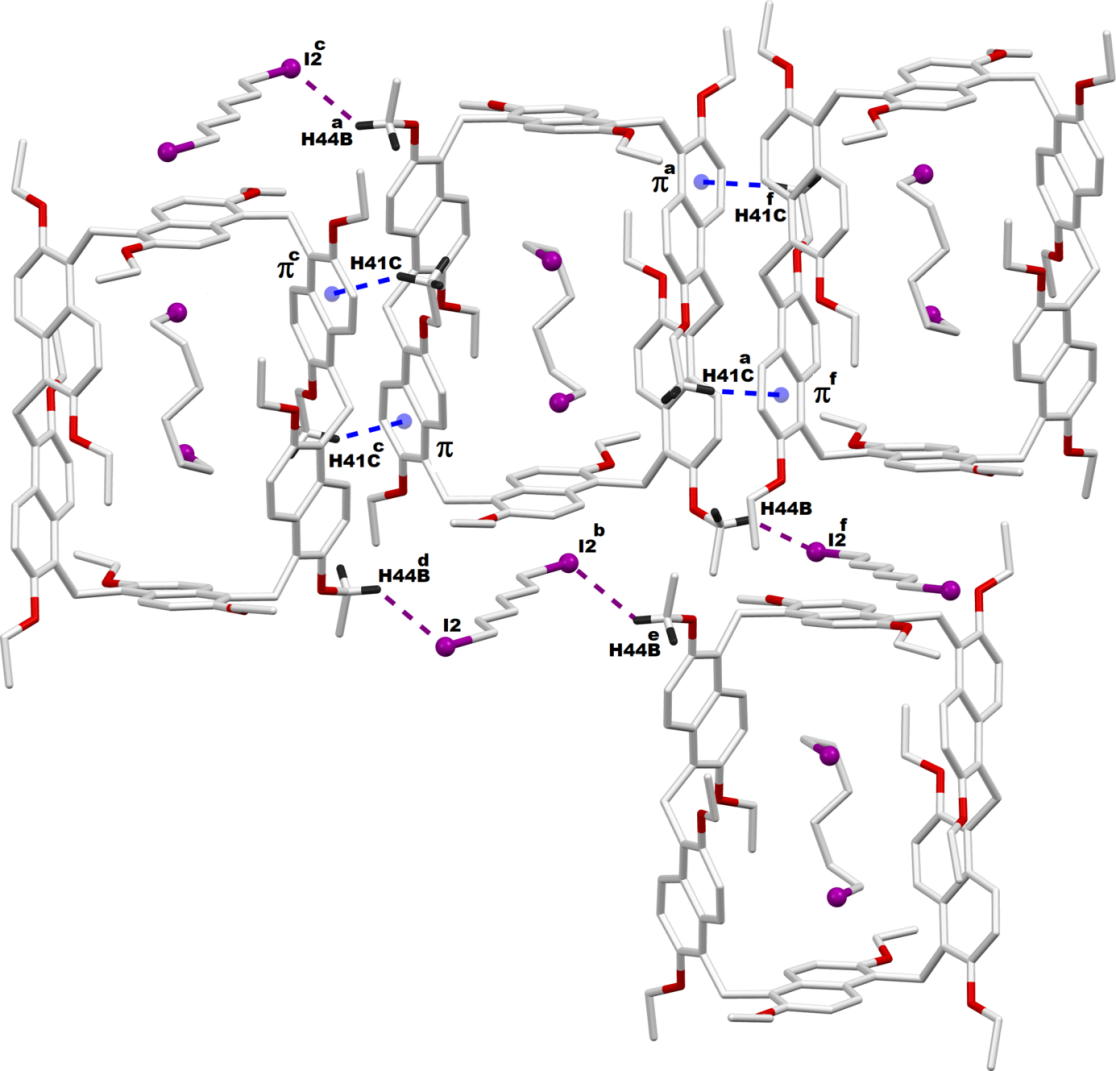
Inter­molecular non-bonding inter­actions among **PS6**–**PS6** and **PS6**–**HexI2** (space-filling solvent). π is the centroid of the C12–C16, C21 phenyl ring. Symmetry code: (*a*) 1 − *x*, *y*, 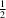 − *z*; (*b*) 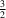 − *x*, 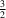 − *y*, 1 − *z*; (*c*) 1 − *x*, 2 − *y*, 1 − *z*; (*d*) *x*, 2 − *y*, 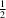 + *z*; (*e*) 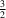 − *x*, −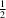 + *y*, 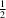 − *z*; (*f*) *x*, 2 − *y*, −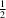 + *z*.

**Figure 6:**
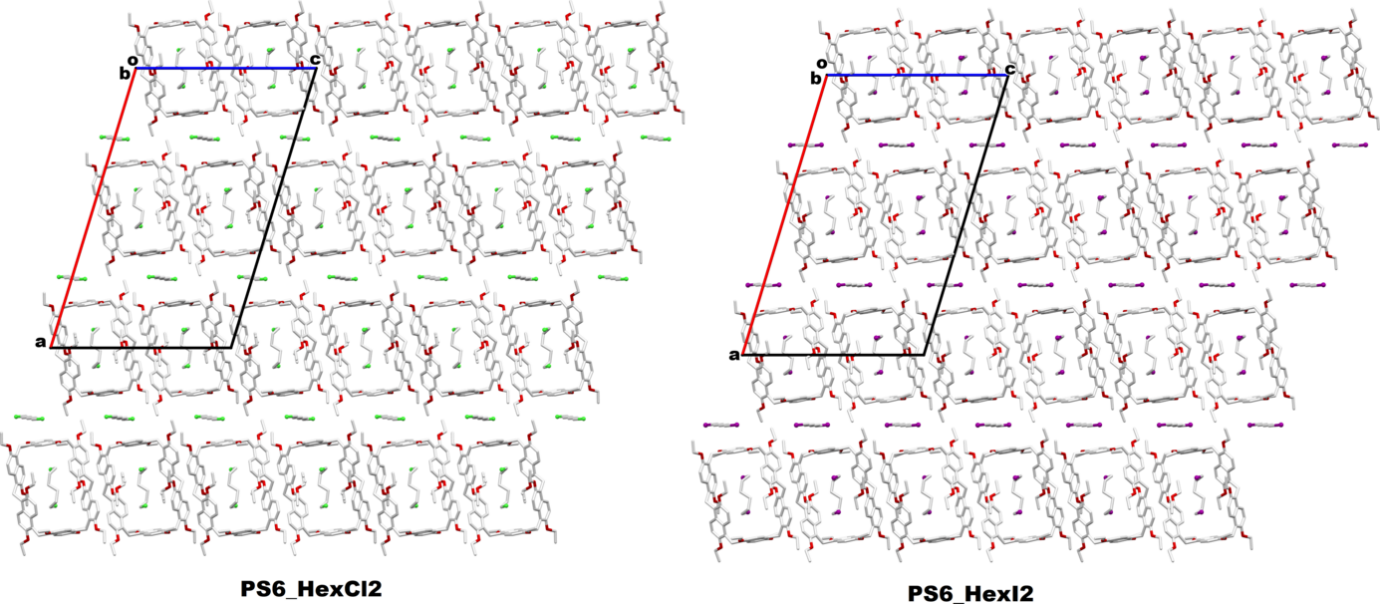
Packing pattern of **PS6·HexCl2** and **PS6·HexI2** systems in the crystal network.

Additionally, in the **PS6·HexI2** crystal packing, inter­molecular C—H⋯π inter­actions are observed among adjacent prism[6]arene macrocycles, as demonstrated in Fig. 5 ▸. The 1,6-di­iodo­hexane mol­ecules present outside the macrocyclic cavity of **PS6·HexI2** are also expected to engage in C—H⋯I inter­actions, which are likewise illustrated in Fig. 5 ▸. The qu­anti­tative details of these inter­molecular non-bonding inter­actions are provided in Table 3 ▸. The packing features of both **PS6·HexCl2** and P**S6·HexI2** crystals are nearly identical and are shown together in Fig. 6 ▸.

## 4. Hirshfeld surface analysis

**Figure 7:**
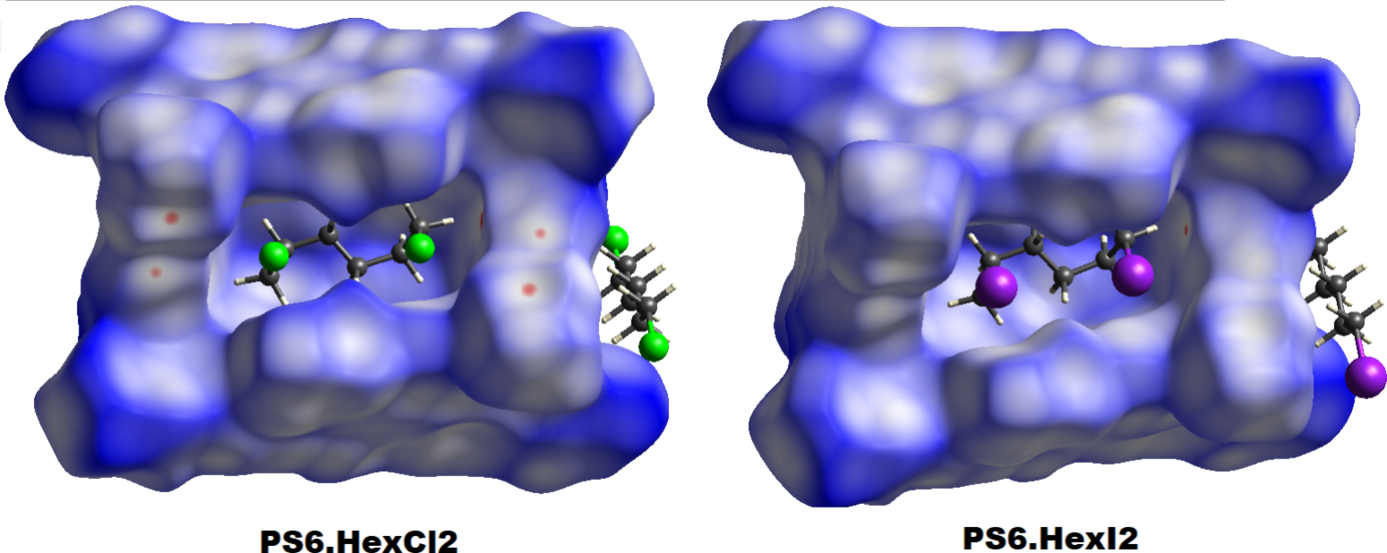
Hirshfeld surfaces (mapped with *d*_norm_) illustrating host–guest inter­actions in **PS6·HexCl2** and **PS6·HexI2**.

Hirshfeld surface analysis using *CrystalExplorer* (Turner *et al.*, 2017 ▸) indicates moderate inter­actions between the prism[6]arene macrocycle and the 1,6-di­chloro­hexa­ne/1,6-di­iodo­hexane present in the cavity. The Hirshfeld surfaces of both **PS6·HexCl2** and **PS6·HexI2** are depicted in Fig. 7 ▸, which shows red spots and white regions inside the macrocyclic cavity corresponding to O⋯H and C—H⋯π bonds, as well as Cl⋯H/I⋯H inter­actions exhibited by the host and guest mol­ecules. These inter­actions collectively contribute to a tighter fit of the guest mol­ecule within the prism[6]arene cavity. The 2D fingerprint plots (McKinnon *et al.*, 2007 ▸) reveal that almost all inter­molecular contacts in these crystal systems involve hydrogen, with the vast majority being H⋯H inter­actions, which account for 74.0% in **PS6·HexCl2** and 73.2% in **PS6·HexI2**, respectively. In the case of **PS6·HexCl2**, the other significant inter­actions are C⋯H (16.2%), Cl⋯H (6.1%), and O⋯H (3.1%), whereas for **PS6·HexI2**, they are C⋯H (16.2%), I⋯H (7.1%), and O⋯H (2.9%). Thus, van der Waals inter­actions play a particularly prominent role in these crystal structures. The slightly higher contribution of I⋯H inter­actions in **PS6·HexI2** compared to the Cl⋯H contribution in **PS6·HexCl2** suggests the presence of non-bonding C—H⋯I inter­actions between the prism[6]arene macrocycle and the 1,6-di­iodo­hexane mol­ecules located outside the macrocyclic cavity, as discussed above.

## 5. Database survey

A search in the Cambridge Structural Database (version 5.46, last update February 2025; Groom *et al.*, 2016 ▸) revealed that the crystal structure of pereth­oxy prism[6]arene has been reported in six different guest/solvent combinations. However, no prism[6]arene macrocycle has been reported encapsulating either 1,6-di­chloro­hexane or 1,6-di­iodo­butane. A structure of pereth­oxy prism[6]arene without a guest mol­ecule inside has been reported, exhibiting a perfect cuboid shape, where all six faces of the cuboid consist of naphthalene moieties. Di­chloro­methane and methanol are present in this crystal as space-filling solvents (TOCQUB; Zhang *et al.*, 2023 ▸). Similarly, pereth­oxy prism[6]arene with no guest inside also retains a perfect cuboid shape when di­chloro­methane alone is present as a space-filling solvent. However, when ethyl acetate is present as the space-filling solvent, the cuboid shape becomes distorted at one end, giving pereth­oxy prism[6]arene a pyramidal shape. In this crystal, the macromolecule does not encapsulate any guest mol­ecule (IVUGUE and IVUGIS; Della Sala *et al.*, 2021 ▸). The crystal structure of meth­oxy prism[6]arene encapsulating a tetra­ethyl­ammonium ion inside the macrocyclic cavity has also been reported (IVUHOZS; Della Sala *et al.*, 2021 ▸). The prism[6]arene in this structure adopts a deformed cuboid shape, almost similar to that of **PS6·HexCl2** or **PS6·HexI2**, with all six faces of the cuboid consisting of naphthalene moieties. A tetra­kis­[3,5-bis­(tri­fluoro­meth­yl)phen­yl]borate anion serves as the counter-anion and is located outside the macrocycle. Additionally, di­chloro­methane is present as another space-filling solvent. A perprop­oxy prism[6]arene with no guest inside the cavity has been reported, exhibiting a slightly deformed cuboid shape, with all six faces of the cuboid consisting of naphthalene moieties. Toluene is present as a space-filling solvent in this crystal (IVUHAL; Della Sala *et al.*, 2021 ▸). Furthermore, an isoprop­oxy prism[6]arene macrocycle has been reported, in which the prism[6]arene adopts a perfect cuboid conformation, with four isopropyl chains folded inside the cavity. These branched isopropyl chains on the prism[6]arene rims engage in C—H⋯π inter­actions with the naphthalene moieties of the macrocycle, thereby filling its inter­nal void and stabilizing the cuboid conformation. Di­chloro­methane and methanol are present as space-filling solvents in this crystal network (RINQIS; Regno *et al.*, 2023 ▸).

## 6. Synthesis and crystallization

Prism[6]arene was synthesized as reported earlier (Della Sala *et al.*, 2021 ▸). Colorless crystals of **PS6·HexCl2** and **PS6·HexI2**, suitable for single-crystal analysis, were grown by dissolving prism[6]arene (10 mg) in a 1,6-di­chloro­methane: 1,6-di­chloro­hexane solvent mixture (90:10 *v/*v, 1 mL) and prism[6]arene (10 mg) in a 1,6-di­chloro­methane: 1,6-di­iodo­hexane solvent mixture (90:10 *v*/*v*, 1 mL), respectively, and subjecting them to slow solvent evaporation.

## 7. Refinement

Crystal data, data collection, and structure refinement details are summarized in Table 4 ▸.

One of the eth­oxy fractions of the prism[6]arene mol­ecule in the **PS6·HexCl2** crystal was found to be disordered. Consequently, refinement of this disordered fraction was carried out using the PART command, with 82.3 (19):17.7 (19) % occupancies for the major and minor components, respectively. The DELU and SIMU commands were used to restrain the thermal factors of these disordered components. Additionally, SIMU and RIGU were used to restrain/constrain the thermal displacement parameters, while the DFIX and DANG commands were applied to adjust the geometry of the 1,6-di­chloro­hexane fragment in this crystal.

For the **PS6·HexI2** crystal, the DELU and SIMU commands were used to restrain the thermal factors of the carbon atoms belonging to the 1,6-di­iodo­hexane fragments. Furthermore, the geometry of one of the eth­oxy components was adjusted using the DFIX command, while SIMU and DELU were used to restrain/constrain the thermal displacement parameters of this fraction.

In both crystals, all hydrogen atoms were positioned geometrically, with C—H distances of 0.96 Å for methyl, 0.97 Å for methyl­ene, and 0.93 Å for aromatic hydrogen atoms. The thermal factors of hydrogen atoms were refined with *U*_iso_(H)=1.2*U*_eq_(C), except for hydrogen atoms from methyl groups, where *U*_iso_(H)=1.5*U*_eq_(C) was applied.

## Supplementary Material

Crystal structure: contains datablock(s) PS6_HexCl2, PS6_HexI2. DOI: 10.1107/S2056989025003767/dx2066sup1.cif

Supporting information file. DOI: 10.1107/S2056989025003767/dx2066PS6_HexCl2sup4.mol

Structure factors: contains datablock(s) PS6_HexCl2. DOI: 10.1107/S2056989025003767/dx2066PS6_HexCl2sup6.hkl

Supporting information file. DOI: 10.1107/S2056989025003767/dx2066PS6_HexI2sup5.mol

Structure factors: contains datablock(s) PS6_HexI2. DOI: 10.1107/S2056989025003767/dx2066PS6_HexI2sup7.hkl

CCDC references: 2431273, 2431272

Additional supporting information:  crystallographic information; 3D view; checkCIF report

## Figures and Tables

**Table 1:** Non-bonding inter­actions between prism[6]arene host and di­chloro­hexane guest (Å, °) π1–π3 are the centroids of the phenyl rings C1–C5,C10, C16–C21 and C23–C27,C32, respectively.

*D*—H⋯*A*	*D*—H	H⋯*A*	*D*⋯*A*	*D*—H⋯*A*
C25—H25⋯Cl1	0.93	3.28	3.776 (5)	116
C34—H34*A*⋯Cl1	0.97	3.45	4.380 (4)	160
C43—H43*C*⋯Cl1	0.96	3.20	3.809 (2)	123
C46—H46*A*⋯O5^i^	0.97	3.19	4.140 (7)	166
C46—H46*B*⋯π1	0.97	3.12	3.926 (6)	145
C47—H47*A*⋯π3	0.97	3.23	4.044 (8)	143
C47—H47*B*⋯π2	0.97	3.32	4.079 (7)	137
C48—H48*A*⋯O4^1^	0.97	3.34	4.278 (7)	162
C48—H48*B*⋯O5	0.97	3.16	3.881 (7)	132

Symmetry code: (i) 1 − *x*, *y*, 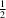 − *z*.

**Table 2:** Non-bonding inter­actions between prism[6]arene host and di­iodo­hexane guest (Å, °) π1–π3 are centroids of the phenyl rings C5–C10, C16–C21 and C23–C27,C32, respectively.

*D*—H⋯*A*	*D*—H	H⋯*A*	*D*⋯*A*	*D*—H⋯*A*
C19—H19⋯I1	0.93	3.38	3.930 (8)	120
C36—H36*A*⋯I1	0.97	3.45	4.385 (6)	163
C41^i^—H41*B*^i^⋯I1	0.96	3.37	3.920 (8)	118
C46—H46*A*⋯O6^i^	0.97	3.07	4.015 (12)	166
C46—H46*B*⋯π1	0.97	3.28	4.019 (8)	135
C47—H47*A*⋯π2	0.97	3.31	4.098 (10)	139
C47—H47*B*⋯π3	0.97	3.32	4.059 (11)	136
C48—H48*A*⋯O3	0.97	3.32	4.271 (10)	165
C48—H48*B*⋯O6	0.97	3.20	3.936 (10)	134

Symmetry code: (i) 1 − *x*, *y*, 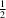 − *z*.

**Table 3:** Inter­molecular non-bonding inter­actions between adjacent prism[6]arenes as well as prism[6]arene and solvent di­iodo­hexane (Å, °) π is the centroid of the C12–C16phenyl ring.

*D*—H⋯*A*	*D*—H	H⋯*A*	*D*⋯*A*	*D*—H⋯*A*
C41—H41*C*⋯π^i^	0.96	2.74	3.492 (8)	136
C44—H44*B*⋯I2^i^	0.97	3.19	3.952 (9)	137

Symmetry code: (i) 1 − *x*, *y*, 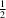 − *z*.

**Table 4:** Experimental details

	**PS6·HexCl2**	**PS6·HexI2**
Crystal data		
Chemical formula	C_90_H_96_O_12_·2C_6_H_12_Cl_2_	C_90_H_96_O_12_·2C_6_H_12_I_2_
*M*_r_	1679.77	2045.57
Crystal system, space group	Monoclinic, *C*2/*c*	Monoclinic, *C*2/*c*
Temperature (K)	293	293
*a*, *b*, *c* (Å)	38.722 (3), 10.4622 (7), 23.8910 (16)	38.728 (3), 10.5427 (6), 24.0234 (16)
β (°)	106.978 (8)	106.814 (8)
*V* (Å^3^)	9256.9 (12)	9389.3 (11)
*Z*	4	4
Radiation type	Mo *K*α	Mo *K*α
μ (mm^−1^)	0.19	1.39
Crystal size (mm)	0.20 × 0.16 × 0.06	0.20 × 0.17 × 0.15

Data collection		
Diffractometer	Rigaku R-AXIS RAPID	Rigaku R-AXIS RAPID
Absorption correction	Multi-scan (*ABSCOR*; Higashi, 1995 ▸)	Multi-scan (*ABSCOR*; Higashi, 1995 ▸)
*T*_min_, *T*_max_	0.546, 0.897	0.452, 0.884
No. of measured, independent and observed [*I* > 2σ(*I*)] reflections	31898, 8092, 4016	27851, 8193, 4349
*R*_int_	0.112	0.077
(sin θ/λ)_max_ (Å^−1^)	0.594	0.595

Refinement		
*R*[*F*^2^ > 2σ(*F*^2^)], *wR*(*F*^2^), *S*	0.071, 0.187, 1.00	0.069, 0.192, 1.02
No. of reflections	8092	8193
No. of parameters	556	532
No. of restraints	92	46
H-atom treatment	H-atom parameters constrained	H-atom parameters constrained
Δρ_max_, Δρ_min_ (e Å^−3^)	0.28, −0.24	0.70, −0.77

Computer programs: *CrystalClear* (Rigaku, 2016 ▸), *CrystalStructure* (Rigaku, 2017 ▸), *SHELXL2019/3* (Sheldrick, 2015 ▸) and *Mercury* (Macrae *et al.*, 2020 ▸).

## supplementary crystallographic information

### Perethoxy-prism[6]arene 1,6-dichlorohexane disolvate (PS6_HexCl2) Crystal data

**Table:**

C_90_H_96_O_12_·2C_6_H_12_Cl_2_	*F*(000) = 3584
*M**_r_* = 1679.77	*D*_x_ = 1.205 Mg m^−^^3^
Monoclinic, *C*2/*c*	Mo *K*α radiation, λ = 0.71075 Å
*a* = 38.722 (3) Å	Cell parameters from 14258 reflections
*b* = 10.4622 (7) Å	θ = 3.2–24.9°
*c* = 23.8910 (16) Å	µ = 0.19 mm^−^^1^
β = 106.978 (8)°	*T* = 293 K
*V* = 9256.9 (12) Å^3^	Block, colorless
*Z* = 4	0.20 × 0.16 × 0.06 mm

### Perethoxy-prism[6]arene 1,6-dichlorohexane disolvate (PS6_HexCl2) Data collection

**Table:**

Rigaku R-AXIS RAPID diffractometer	4016 reflections with *I* > 2σ(*I*)
Detector resolution: 10.000 pixels mm^-1^	*R*_int_ = 0.112
ω scans	θ_max_ = 25.0°, θ_min_ = 3.2°
Absorption correction: multi-scan (ABSCOR; Higashi, 1995)	*h* = −42→45
*T*_min_ = 0.546, *T*_max_ = 0.897	*k* = −12→12
31898 measured reflections	*l* = −28→28
8092 independent reflections	

### Perethoxy-prism[6]arene 1,6-dichlorohexane disolvate (PS6_HexCl2) Refinement

**Table:**

Refinement on *F*^2^	Primary atom site location: structure-invariant direct methods
Least-squares matrix: full	Hydrogen site location: inferred from neighbouring sites
*R*[*F*^2^ > 2σ(*F*^2^)] = 0.071	H-atom parameters constrained
*wR*(*F*^2^) = 0.187	*w* = 1/[σ^2^(*F*_o_^2^) + (0.0844*P*)^2^] where *P* = (*F*_o_^2^ + 2*F*_c_^2^)/3
*S* = 1.00	(Δ/σ)_max_ < 0.001
8092 reflections	Δρ_max_ = 0.28 e Å^−^^3^
556 parameters	Δρ_min_ = −0.24 e Å^−^^3^
92 restraints	

### Perethoxy-prism[6]arene 1,6-dichlorohexane disolvate (PS6_HexCl2) Special details

**Table:**

Geometry. All esds (except the esd in the dihedral angle between two l.s. planes) are estimated using the full covariance matrix. The cell esds are taken into account individually in the estimation of esds in distances, angles and torsion angles; correlations between esds in cell parameters are only used when they are defined by crystal symmetry. An approximate (isotropic) treatment of cell esds is used for estimating esds involving l.s. planes.

### Perethoxy-prism[6]arene 1,6-dichlorohexane disolvate (PS6_HexCl2) Fractional atomic coordinates and isotropic or equivalent isotropic displacement parameters (Å^2^)

**Table:**

	*x*	*y*	*z*	*U*_iso_*/*U*_eq_	Occ. (<1)
Cl1	0.56472 (5)	0.59286 (18)	0.29326 (8)	0.1364 (6)	
O1	0.66007 (7)	0.5601 (2)	0.46893 (11)	0.0558 (7)	
C1	0.65386 (8)	0.3419 (3)	0.44409 (15)	0.0384 (8)	
O2	0.66298 (7)	−0.0535 (2)	0.28958 (11)	0.0579 (7)	
C2	0.66023 (9)	0.4651 (3)	0.42885 (15)	0.0415 (9)	
Cl2	0.75364 (4)	0.66424 (12)	0.57427 (7)	0.1020 (5)	
O3	0.68655 (7)	0.4117 (3)	0.20064 (12)	0.0676 (8)	
C3	0.66764 (10)	0.4906 (3)	0.37609 (16)	0.0476 (9)	
H3	0.672201	0.574234	0.367057	0.057*	
C4	0.66830 (9)	0.3950 (3)	0.33767 (16)	0.0472 (9)	
H4	0.673150	0.414788	0.302763	0.057*	
O5	0.47899 (6)	0.4852 (2)	0.40860 (12)	0.0580 (7)	
C5	0.66178 (8)	0.2668 (3)	0.34972 (15)	0.0390 (8)	
C6	0.66318 (9)	0.1658 (3)	0.31055 (15)	0.0409 (9)	
O6	0.64885 (7)	0.0852 (3)	0.56017 (12)	0.0671 (8)	
C7	0.65982 (9)	0.0426 (3)	0.32739 (16)	0.0457 (9)	
C10	0.65495 (8)	0.2409 (3)	0.40455 (15)	0.0384 (8)	
C9	0.65053 (9)	0.1118 (3)	0.41814 (16)	0.0461 (9)	
H9	0.645875	0.091879	0.453179	0.055*	
C8	0.65293 (10)	0.0156 (3)	0.38086 (17)	0.0504 (10)	
H8	0.649992	−0.068679	0.390899	0.061*	
C12	0.64242 (9)	0.2576 (3)	0.20262 (15)	0.0430 (9)	
C11	0.67070 (9)	0.1915 (3)	0.25260 (15)	0.0472 (9)	
H11A	0.676156	0.109986	0.237826	0.057*	
H11B	0.692539	0.242525	0.261022	0.057*	
C13	0.65262 (9)	0.3629 (4)	0.17603 (16)	0.0490 (10)	
C15	0.59554 (10)	0.3656 (4)	0.10091 (17)	0.0534 (10)	
H15	0.580666	0.402366	0.066958	0.064*	
C14	0.62879 (10)	0.4144 (4)	0.12512 (17)	0.0565 (11)	
H14	0.636150	0.484363	0.107480	0.068*	
C16	0.58271 (9)	0.2596 (3)	0.12598 (15)	0.0426 (9)	
C17	0.54758 (9)	0.2068 (3)	0.10158 (15)	0.0450 (9)	
C18	0.53695 (9)	0.1050 (3)	0.12930 (17)	0.0476 (9)	
C20	0.59445 (10)	0.1009 (4)	0.20321 (16)	0.0519 (10)	
H20	0.609824	0.062348	0.236216	0.062*	
C19	0.56058 (10)	0.0530 (4)	0.18043 (18)	0.0579 (11)	
H19	0.552971	−0.014967	0.198969	0.069*	
C21	0.60705 (9)	0.2069 (3)	0.17852 (15)	0.0419 (9)	
C22	0.52281 (9)	0.2503 (4)	0.04232 (15)	0.0493 (10)	
H22A	0.535923	0.311378	0.025730	0.059*	
H22B	0.517556	0.176867	0.016425	0.059*	
C24	0.51238 (10)	0.4320 (3)	0.43228 (15)	0.0459 (9)	
C23	0.51300 (9)	0.3112 (3)	0.45722 (15)	0.0440 (9)	
C27	0.57991 (9)	0.3132 (3)	0.48233 (14)	0.0395 (8)	
C26	0.57686 (10)	0.4357 (3)	0.45583 (15)	0.0449 (9)	
H26	0.597767	0.477681	0.454480	0.054*	
C25	0.54456 (10)	0.4935 (3)	0.43243 (16)	0.0468 (9)	
H25	0.543728	0.574720	0.416301	0.056*	
C30	0.58361 (10)	0.0823 (4)	0.54138 (17)	0.0537 (10)	
H30	0.585058	0.007079	0.562668	0.064*	
C29	0.61525 (10)	0.1394 (4)	0.53691 (16)	0.0485 (9)	
C28	0.61390 (9)	0.2544 (3)	0.50677 (15)	0.0416 (9)	
C40	0.70105 (12)	0.4994 (5)	0.1687 (2)	0.0822 (15)	
H40A	0.686704	0.576914	0.161429	0.099*	
H40B	0.700923	0.462680	0.131394	0.099*	
O4	0.50337 (7)	0.0539 (2)	0.10384 (13)	0.0658 (8)	
C38A	0.4882 (2)	−0.0298 (11)	0.1382 (4)	0.091 (3)	0.823 (19)
H38A	0.492301	0.005154	0.177188	0.109*	0.823 (19)
H38B	0.499786	−0.112834	0.141696	0.109*	0.823 (19)
C39A	0.4488 (3)	−0.0440 (16)	0.1097 (7)	0.154 (6)	0.823 (19)
H39A	0.438738	−0.099904	0.132620	0.231*	0.823 (19)
H39B	0.437433	0.038211	0.106636	0.231*	0.823 (19)
H39C	0.444898	−0.079474	0.071236	0.231*	0.823 (19)
C38B	0.4787 (7)	0.041 (4)	0.1381 (13)	0.074 (8)	0.177 (19)
H38C	0.461905	0.111628	0.131336	0.089*	0.177 (19)
H38D	0.491575	0.036377	0.179484	0.089*	0.177 (19)
C39B	0.4593 (12)	−0.082 (3)	0.118 (3)	0.091 (12)	0.177 (19)
H39D	0.442150	−0.097576	0.138746	0.136*	0.177 (19)
H39E	0.446972	−0.076001	0.076526	0.136*	0.177 (19)
H39F	0.476442	−0.150743	0.124349	0.136*	0.177 (19)
C37	0.65758 (16)	−0.2586 (4)	0.2485 (2)	0.1072 (19)	
H37A	0.650488	−0.345044	0.252785	0.161*	
H37B	0.682274	−0.257160	0.247908	0.161*	
H37C	0.642414	−0.223880	0.212443	0.161*	
C36	0.65381 (14)	−0.1801 (4)	0.29858 (19)	0.0790 (14)	
H36A	0.629178	−0.184071	0.300598	0.095*	
H36B	0.669731	−0.212431	0.335087	0.095*	
C35	0.64472 (17)	0.7641 (4)	0.4955 (2)	0.108 (2)	
H35A	0.643353	0.853238	0.485541	0.161*	
H35B	0.663125	0.751231	0.531924	0.161*	
H35C	0.621878	0.736144	0.499213	0.161*	
C34	0.65372 (12)	0.6891 (3)	0.44855 (19)	0.0677 (12)	
H34A	0.633875	0.692308	0.412696	0.081*	
H34B	0.675091	0.723778	0.440774	0.081*	
C33	0.64855 (9)	0.3169 (3)	0.50395 (14)	0.0401 (9)	
H33A	0.668445	0.263923	0.525957	0.048*	
H33B	0.650637	0.398196	0.524205	0.048*	
C32	0.54691 (9)	0.2518 (3)	0.48389 (15)	0.0431 (9)	
C31	0.55082 (10)	0.1343 (3)	0.51531 (16)	0.0503 (10)	
H31	0.530230	0.092106	0.517997	0.060*	
C42	0.47697 (10)	0.6130 (4)	0.38585 (18)	0.0582 (11)	
H42A	0.487362	0.616032	0.353526	0.070*	
H42B	0.490389	0.671011	0.416073	0.070*	
C41	0.73890 (14)	0.5289 (6)	0.2042 (2)	0.121 (2)	
H41A	0.749934	0.584029	0.182258	0.182*	
H41B	0.752453	0.450936	0.213341	0.182*	
H41C	0.738602	0.570720	0.239849	0.182*	
C43	0.43802 (11)	0.6519 (4)	0.3653 (2)	0.0727 (13)	
H43A	0.436186	0.737654	0.350444	0.109*	
H43B	0.427902	0.647991	0.397504	0.109*	
H43C	0.425036	0.595007	0.334930	0.109*	
C46	0.56990 (18)	0.4348 (6)	0.2688 (3)	0.146 (3)	
H46A	0.561218	0.433492	0.226397	0.175*	
H46B	0.595433	0.414268	0.280079	0.175*	
C45	0.69531 (13)	−0.0236 (6)	0.6281 (2)	0.112 (2)	
H45A	0.700078	−0.088174	0.657956	0.168*	
H45B	0.708396	0.052689	0.643610	0.168*	
H45C	0.702857	−0.053650	0.595564	0.168*	
C44	0.65571 (13)	0.0049 (5)	0.60819 (19)	0.0855 (15)	
H44A	0.642098	−0.073782	0.597683	0.103*	
H44B	0.648476	0.045697	0.639444	0.103*	
C47	0.55092 (19)	0.3345 (5)	0.2916 (3)	0.142 (2)	
H47A	0.561860	0.329454	0.333579	0.171*	
H47B	0.555356	0.253517	0.275156	0.171*	
C48	0.51000 (19)	0.3486 (6)	0.2801 (2)	0.134 (2)	
H48A	0.501464	0.279013	0.299374	0.161*	
H48B	0.505258	0.427828	0.297572	0.161*	
C49	0.75588 (12)	0.5492 (4)	0.5202 (2)	0.0775 (13)	
H49A	0.737641	0.569163	0.483734	0.093*	
H49B	0.779309	0.554796	0.513270	0.093*	
C50	0.75018 (11)	0.4155 (4)	0.5381 (2)	0.0656 (12)	
H50A	0.726613	0.409246	0.544408	0.079*	
H50B	0.768259	0.395474	0.574659	0.079*	
C51	0.75267 (11)	0.3188 (3)	0.4914 (2)	0.0637 (11)	
H51A	0.734467	0.338918	0.454969	0.076*	
H51B	0.776137	0.326242	0.484808	0.076*	

### Perethoxy-prism[6]arene 1,6-dichlorohexane disolvate (PS6_HexCl2) Atomic displacement parameters (Å^2^)

**Table:**

	*U*^11^	*U*^22^	*U*^33^	*U*^12^	*U*^13^	*U*^23^
Cl1	0.1564 (16)	0.1473 (15)	0.1135 (13)	−0.0262 (12)	0.0518 (12)	−0.0044 (12)
O1	0.0794 (19)	0.0372 (15)	0.0552 (17)	−0.0047 (12)	0.0266 (14)	−0.0015 (13)
C1	0.0360 (19)	0.042 (2)	0.037 (2)	0.0002 (15)	0.0098 (16)	0.0022 (18)
O2	0.082 (2)	0.0433 (16)	0.0501 (16)	0.0053 (13)	0.0222 (14)	−0.0016 (14)
C2	0.049 (2)	0.035 (2)	0.041 (2)	0.0003 (16)	0.0141 (17)	−0.0005 (18)
Cl2	0.1175 (12)	0.0737 (8)	0.1200 (12)	−0.0091 (7)	0.0429 (9)	−0.0068 (8)
O3	0.0536 (17)	0.087 (2)	0.0581 (18)	−0.0188 (15)	0.0091 (14)	0.0205 (16)
C3	0.064 (3)	0.032 (2)	0.049 (2)	−0.0042 (17)	0.0204 (19)	0.0078 (19)
C4	0.061 (2)	0.043 (2)	0.040 (2)	−0.0003 (18)	0.0190 (18)	0.008 (2)
O5	0.0463 (16)	0.0552 (16)	0.0690 (19)	0.0048 (13)	0.0114 (14)	0.0084 (14)
C5	0.035 (2)	0.042 (2)	0.039 (2)	0.0023 (15)	0.0093 (16)	0.0055 (18)
C6	0.040 (2)	0.041 (2)	0.041 (2)	0.0044 (16)	0.0105 (17)	0.0039 (18)
O6	0.0591 (18)	0.0735 (19)	0.0720 (19)	0.0137 (14)	0.0241 (15)	0.0383 (16)
C7	0.051 (2)	0.047 (2)	0.039 (2)	0.0070 (17)	0.0113 (18)	0.000 (2)
C10	0.036 (2)	0.041 (2)	0.037 (2)	0.0002 (15)	0.0086 (16)	0.0061 (18)
C9	0.056 (2)	0.045 (2)	0.040 (2)	−0.0002 (17)	0.0174 (18)	0.0021 (19)
C8	0.066 (3)	0.032 (2)	0.052 (2)	−0.0003 (17)	0.016 (2)	0.006 (2)
C12	0.044 (2)	0.049 (2)	0.039 (2)	0.0046 (17)	0.0168 (17)	0.0003 (19)
C11	0.047 (2)	0.050 (2)	0.045 (2)	0.0076 (17)	0.0145 (18)	0.0045 (19)
C13	0.040 (2)	0.065 (3)	0.045 (2)	−0.0018 (19)	0.0152 (18)	0.011 (2)
C15	0.046 (2)	0.068 (3)	0.046 (2)	0.003 (2)	0.0134 (19)	0.017 (2)
C14	0.051 (3)	0.069 (3)	0.053 (2)	0.001 (2)	0.019 (2)	0.027 (2)
C16	0.042 (2)	0.049 (2)	0.041 (2)	0.0036 (17)	0.0187 (17)	0.0034 (19)
C17	0.045 (2)	0.050 (2)	0.043 (2)	0.0093 (18)	0.0178 (18)	0.0012 (19)
C18	0.040 (2)	0.044 (2)	0.059 (3)	0.0008 (18)	0.0141 (19)	−0.001 (2)
C20	0.053 (2)	0.053 (2)	0.045 (2)	0.0017 (19)	0.0072 (19)	0.008 (2)
C19	0.057 (3)	0.053 (2)	0.063 (3)	−0.002 (2)	0.016 (2)	0.015 (2)
C21	0.047 (2)	0.042 (2)	0.040 (2)	0.0069 (17)	0.0173 (18)	0.0056 (18)
C22	0.043 (2)	0.063 (2)	0.041 (2)	0.0040 (18)	0.0124 (17)	−0.001 (2)
C24	0.045 (2)	0.052 (2)	0.040 (2)	0.0049 (18)	0.0114 (18)	−0.0011 (18)
C23	0.044 (2)	0.051 (2)	0.038 (2)	−0.0048 (17)	0.0139 (17)	−0.0068 (19)
C27	0.043 (2)	0.044 (2)	0.0330 (19)	−0.0028 (16)	0.0125 (16)	−0.0017 (17)
C26	0.043 (2)	0.046 (2)	0.047 (2)	−0.0028 (17)	0.0160 (18)	0.0006 (19)
C25	0.046 (2)	0.048 (2)	0.049 (2)	−0.0033 (18)	0.0163 (18)	0.0033 (19)
C30	0.057 (3)	0.050 (2)	0.057 (3)	−0.001 (2)	0.023 (2)	0.016 (2)
C29	0.048 (2)	0.057 (2)	0.043 (2)	0.0053 (19)	0.0179 (19)	0.007 (2)
C28	0.045 (2)	0.043 (2)	0.037 (2)	−0.0023 (17)	0.0130 (17)	0.0002 (18)
C40	0.072 (3)	0.111 (4)	0.066 (3)	−0.033 (3)	0.024 (3)	0.002 (3)
O4	0.0505 (17)	0.0667 (18)	0.076 (2)	−0.0090 (14)	0.0121 (15)	0.0088 (16)
C38A	0.068 (5)	0.078 (6)	0.117 (5)	−0.029 (4)	0.014 (4)	0.016 (5)
C39A	0.074 (6)	0.191 (13)	0.183 (10)	−0.040 (8)	0.015 (6)	0.081 (10)
C38B	0.020 (12)	0.086 (17)	0.109 (17)	−0.001 (12)	0.008 (10)	0.010 (16)
C39B	0.09 (2)	0.038 (13)	0.15 (3)	0.010 (12)	0.05 (2)	0.030 (17)
C37	0.192 (6)	0.056 (3)	0.084 (4)	−0.013 (3)	0.055 (4)	−0.018 (3)
C36	0.138 (4)	0.042 (2)	0.060 (3)	−0.006 (2)	0.033 (3)	−0.010 (2)
C35	0.193 (6)	0.052 (3)	0.106 (4)	0.015 (3)	0.087 (4)	0.002 (3)
C34	0.104 (4)	0.037 (2)	0.072 (3)	0.001 (2)	0.041 (3)	0.003 (2)
C33	0.043 (2)	0.042 (2)	0.035 (2)	0.0000 (16)	0.0112 (16)	0.0022 (17)
C32	0.046 (2)	0.050 (2)	0.034 (2)	−0.0037 (17)	0.0132 (17)	0.0001 (18)
C31	0.050 (2)	0.049 (2)	0.053 (2)	−0.0102 (18)	0.0171 (19)	0.004 (2)
C42	0.057 (3)	0.057 (3)	0.058 (3)	0.012 (2)	0.013 (2)	0.004 (2)
C41	0.076 (4)	0.176 (6)	0.103 (4)	−0.059 (4)	0.013 (3)	0.002 (4)
C43	0.073 (3)	0.067 (3)	0.071 (3)	0.008 (2)	0.010 (2)	−0.002 (2)
C46	0.129 (6)	0.144 (6)	0.177 (7)	0.038 (5)	0.063 (5)	0.003 (6)
C45	0.077 (4)	0.177 (6)	0.077 (4)	0.031 (4)	0.015 (3)	0.058 (4)
C44	0.079 (3)	0.123 (4)	0.055 (3)	0.021 (3)	0.022 (3)	0.036 (3)
C47	0.167 (6)	0.118 (5)	0.146 (6)	0.033 (5)	0.053 (5)	−0.005 (5)
C48	0.175 (6)	0.134 (5)	0.100 (5)	−0.012 (5)	0.051 (5)	−0.005 (4)
C49	0.074 (3)	0.071 (3)	0.086 (4)	0.000 (2)	0.021 (3)	0.003 (3)
C50	0.048 (2)	0.067 (3)	0.079 (3)	0.000 (2)	0.015 (2)	0.003 (3)
C51	0.044 (2)	0.069 (3)	0.076 (3)	0.003 (2)	0.015 (2)	0.007 (3)

### Perethoxy-prism[6]arene 1,6-dichlorohexane disolvate (PS6_HexCl2) Geometric parameters (Å, º)

**Table:**

Cl1—C46	1.784 (6)	C40—C41	1.495 (6)
O1—C2	1.382 (4)	C40—H40A	0.9700
O1—C34	1.431 (4)	C40—H40B	0.9700
C1—C2	1.381 (4)	O4—C38B	1.435 (17)
C1—C10	1.426 (5)	O4—C38A	1.439 (7)
C1—C33	1.526 (4)	C38A—C39A	1.483 (11)
O2—C7	1.381 (4)	C38A—H38A	0.9700
O2—C36	1.404 (4)	C38A—H38B	0.9700
C2—C3	1.398 (5)	C39A—H39A	0.9600
Cl2—C49	1.787 (5)	C39A—H39B	0.9600
O3—C13	1.371 (4)	C39A—H39C	0.9600
O3—C40	1.410 (5)	C38B—C39B	1.492 (19)
C3—C4	1.363 (5)	C38B—H38C	0.9700
C3—H3	0.9300	C38B—H38D	0.9700
C4—C5	1.410 (4)	C39B—H39D	0.9600
C4—H4	0.9300	C39B—H39E	0.9600
O5—C24	1.369 (4)	C39B—H39F	0.9600
O5—C42	1.436 (4)	C37—C36	1.494 (6)
C5—C6	1.423 (5)	C37—H37A	0.9600
C5—C10	1.436 (4)	C37—H37B	0.9600
C6—C7	1.368 (5)	C37—H37C	0.9600
C6—C11	1.520 (5)	C36—H36A	0.9700
O6—C29	1.379 (4)	C36—H36B	0.9700
O6—C44	1.384 (5)	C35—C34	1.491 (6)
C7—C8	1.407 (5)	C35—H35A	0.9600
C10—C9	1.411 (5)	C35—H35B	0.9600
C9—C8	1.365 (5)	C35—H35C	0.9600
C9—H9	0.9300	C34—H34A	0.9700
C8—H8	0.9300	C34—H34B	0.9700
C12—C13	1.386 (5)	C33—H33A	0.9700
C12—C21	1.424 (5)	C33—H33B	0.9700
C12—C11	1.529 (5)	C32—C31	1.425 (5)
C11—H11A	0.9700	C31—H31	0.9300
C11—H11B	0.9700	C42—C43	1.500 (5)
C13—C14	1.402 (5)	C42—H42A	0.9700
C15—C14	1.348 (5)	C42—H42B	0.9700
C15—C16	1.417 (5)	C41—H41A	0.9600
C15—H15	0.9300	C41—H41B	0.9600
C14—H14	0.9300	C41—H41C	0.9600
C16—C17	1.426 (5)	C43—H43A	0.9600
C16—C21	1.441 (5)	C43—H43B	0.9600
C17—C18	1.379 (5)	C43—H43C	0.9600
C17—C22	1.529 (5)	C46—C47	1.473 (6)
C18—O4	1.373 (4)	C46—H46A	0.9700
C18—C19	1.404 (5)	C46—H46B	0.9700
C20—C19	1.361 (5)	C45—C44	1.497 (6)
C20—C21	1.408 (5)	C45—H45A	0.9600
C20—H20	0.9300	C45—H45B	0.9600
C19—H19	0.9300	C45—H45C	0.9600
C22—C23^i^	1.529 (5)	C44—H44A	0.9700
C22—H22A	0.9700	C44—H44B	0.9700
C22—H22B	0.9700	C47—C48	1.534 (7)
C24—C23	1.395 (5)	C47—H47A	0.9700
C24—C25	1.402 (5)	C47—H47B	0.9700
C23—C32	1.424 (5)	C48—C48^i^	1.419 (11)
C27—C28	1.416 (5)	C48—H48A	0.9700
C27—C26	1.418 (5)	C48—H48B	0.9700
C27—C32	1.440 (5)	C49—C50	1.498 (5)
C26—C25	1.354 (5)	C49—H49A	0.9700
C26—H26	0.9300	C49—H49B	0.9700
C25—H25	0.9300	C50—C51	1.529 (6)
C30—C31	1.354 (5)	C50—H50A	0.9700
C30—C29	1.395 (5)	C50—H50B	0.9700
C30—H30	0.9300	C51—C51^ii^	1.526 (7)
C29—C28	1.395 (5)	C51—H51A	0.9700
C28—C33	1.512 (4)	C51—H51B	0.9700
			
C2—O1—C34	118.5 (3)	C38A—C39A—H39C	109.5
C2—C1—C10	118.3 (3)	H39A—C39A—H39C	109.5
C2—C1—C33	119.4 (3)	H39B—C39A—H39C	109.5
C10—C1—C33	122.1 (3)	O4—C38B—C39B	105 (2)
C7—O2—C36	120.8 (3)	O4—C38B—H38C	110.8
C1—C2—O1	116.8 (3)	C39B—C38B—H38C	110.8
C1—C2—C3	121.0 (3)	O4—C38B—H38D	110.8
O1—C2—C3	122.1 (3)	C39B—C38B—H38D	110.8
C13—O3—C40	119.5 (3)	H38C—C38B—H38D	108.9
C4—C3—C2	121.1 (3)	C38B—C39B—H39D	109.5
C4—C3—H3	119.5	C38B—C39B—H39E	109.5
C2—C3—H3	119.5	H39D—C39B—H39E	109.5
C3—C4—C5	121.4 (3)	C38B—C39B—H39F	109.5
C3—C4—H4	119.3	H39D—C39B—H39F	109.5
C5—C4—H4	119.3	H39E—C39B—H39F	109.5
C24—O5—C42	118.4 (3)	C36—C37—H37A	109.5
C4—C5—C6	121.9 (3)	C36—C37—H37B	109.5
C4—C5—C10	117.2 (3)	H37A—C37—H37B	109.5
C6—C5—C10	120.8 (3)	C36—C37—H37C	109.5
C7—C6—C5	118.8 (3)	H37A—C37—H37C	109.5
C7—C6—C11	119.7 (3)	H37B—C37—H37C	109.5
C5—C6—C11	121.5 (3)	O2—C36—C37	107.9 (4)
C29—O6—C44	121.0 (3)	O2—C36—H36A	110.1
C6—C7—O2	117.3 (3)	C37—C36—H36A	110.1
C6—C7—C8	121.0 (3)	O2—C36—H36B	110.1
O2—C7—C8	121.6 (3)	C37—C36—H36B	110.1
C9—C10—C1	121.9 (3)	H36A—C36—H36B	108.4
C9—C10—C5	117.2 (3)	C34—C35—H35A	109.5
C1—C10—C5	120.9 (3)	C34—C35—H35B	109.5
C8—C9—C10	121.3 (3)	H35A—C35—H35B	109.5
C8—C9—H9	119.4	C34—C35—H35C	109.5
C10—C9—H9	119.4	H35A—C35—H35C	109.5
C9—C8—C7	120.8 (3)	H35B—C35—H35C	109.5
C9—C8—H8	119.6	O1—C34—C35	107.2 (3)
C7—C8—H8	119.6	O1—C34—H34A	110.3
C13—C12—C21	118.7 (3)	C35—C34—H34A	110.3
C13—C12—C11	118.8 (3)	O1—C34—H34B	110.3
C21—C12—C11	122.2 (3)	C35—C34—H34B	110.3
C6—C11—C12	120.5 (3)	H34A—C34—H34B	108.5
C6—C11—H11A	107.2	C28—C33—C1	118.7 (3)
C12—C11—H11A	107.2	C28—C33—H33A	107.6
C6—C11—H11B	107.2	C1—C33—H33A	107.6
C12—C11—H11B	107.2	C28—C33—H33B	107.6
H11A—C11—H11B	106.8	C1—C33—H33B	107.6
O3—C13—C12	117.7 (3)	H33A—C33—H33B	107.1
O3—C13—C14	122.3 (3)	C23—C32—C31	123.6 (3)
C12—C13—C14	120.0 (3)	C23—C32—C27	120.2 (3)
C14—C15—C16	121.6 (3)	C31—C32—C27	116.1 (3)
C14—C15—H15	119.2	C30—C31—C32	122.0 (3)
C16—C15—H15	119.2	C30—C31—H31	119.0
C15—C14—C13	122.1 (4)	C32—C31—H31	119.0
C15—C14—H14	119.0	O5—C42—C43	108.4 (3)
C13—C14—H14	119.0	O5—C42—H42A	110.0
C15—C16—C17	122.7 (3)	C43—C42—H42A	110.0
C15—C16—C21	116.5 (3)	O5—C42—H42B	110.0
C17—C16—C21	120.7 (3)	C43—C42—H42B	110.0
C18—C17—C16	119.1 (3)	H42A—C42—H42B	108.4
C18—C17—C22	118.7 (3)	C40—C41—H41A	109.5
C16—C17—C22	121.9 (3)	C40—C41—H41B	109.5
O4—C18—C17	117.7 (3)	H41A—C41—H41B	109.5
O4—C18—C19	121.8 (3)	C40—C41—H41C	109.5
C17—C18—C19	120.4 (3)	H41A—C41—H41C	109.5
C19—C20—C21	122.3 (3)	H41B—C41—H41C	109.5
C19—C20—H20	118.9	C42—C43—H43A	109.5
C21—C20—H20	118.9	C42—C43—H43B	109.5
C20—C19—C18	120.8 (4)	H43A—C43—H43B	109.5
C20—C19—H19	119.6	C42—C43—H43C	109.5
C18—C19—H19	119.6	H43A—C43—H43C	109.5
C20—C21—C12	122.3 (3)	H43B—C43—H43C	109.5
C20—C21—C16	116.6 (3)	C47—C46—Cl1	115.0 (5)
C12—C21—C16	121.1 (3)	C47—C46—H46A	108.5
C23^i^—C22—C17	116.3 (3)	Cl1—C46—H46A	108.5
C23^i^—C22—H22A	108.2	C47—C46—H46B	108.5
C17—C22—H22A	108.2	Cl1—C46—H46B	108.5
C23^i^—C22—H22B	108.2	H46A—C46—H46B	107.5
C17—C22—H22B	108.2	C44—C45—H45A	109.5
H22A—C22—H22B	107.4	C44—C45—H45B	109.5
O5—C24—C23	116.2 (3)	H45A—C45—H45B	109.5
O5—C24—C25	123.1 (3)	C44—C45—H45C	109.5
C23—C24—C25	120.7 (3)	H45A—C45—H45C	109.5
C24—C23—C32	119.0 (3)	H45B—C45—H45C	109.5
C24—C23—C22^i^	118.6 (3)	O6—C44—C45	108.4 (4)
C32—C23—C22^i^	122.4 (3)	O6—C44—H44A	110.0
C28—C27—C26	121.5 (3)	C45—C44—H44A	110.0
C28—C27—C32	121.4 (3)	O6—C44—H44B	110.0
C26—C27—C32	117.1 (3)	C45—C44—H44B	110.0
C25—C26—C27	122.3 (3)	H44A—C44—H44B	108.4
C25—C26—H26	118.9	C46—C47—C48	117.8 (6)
C27—C26—H26	118.9	C46—C47—H47A	107.9
C26—C25—C24	120.6 (4)	C48—C47—H47A	107.9
C26—C25—H25	119.7	C46—C47—H47B	107.9
C24—C25—H25	119.7	C48—C47—H47B	107.9
C31—C30—C29	121.3 (4)	H47A—C47—H47B	107.2
C31—C30—H30	119.4	C48^i^—C48—C47	114.3 (8)
C29—C30—H30	119.4	C48^i^—C48—H48A	108.7
O6—C29—C28	116.9 (3)	C47—C48—H48A	108.7
O6—C29—C30	122.6 (3)	C48^i^—C48—H48B	108.7
C28—C29—C30	120.5 (3)	C47—C48—H48B	108.7
C29—C28—C27	118.6 (3)	H48A—C48—H48B	107.6
C29—C28—C33	119.8 (3)	C50—C49—Cl2	112.3 (3)
C27—C28—C33	121.6 (3)	C50—C49—H49A	109.1
O3—C40—C41	107.9 (4)	Cl2—C49—H49A	109.1
O3—C40—H40A	110.1	C50—C49—H49B	109.1
C41—C40—H40A	110.1	Cl2—C49—H49B	109.1
O3—C40—H40B	110.1	H49A—C49—H49B	107.9
C41—C40—H40B	110.1	C49—C50—C51	111.4 (4)
H40A—C40—H40B	108.4	C49—C50—H50A	109.4
C18—O4—C38B	119.2 (12)	C51—C50—H50A	109.4
C18—O4—C38A	118.5 (4)	C49—C50—H50B	109.4
O4—C38A—C39A	109.6 (8)	C51—C50—H50B	109.4
O4—C38A—H38A	109.8	H50A—C50—H50B	108.0
C39A—C38A—H38A	109.8	C51^ii^—C51—C50	112.7 (5)
O4—C38A—H38B	109.8	C51^ii^—C51—H51A	109.1
C39A—C38A—H38B	109.8	C50—C51—H51A	109.1
H38A—C38A—H38B	108.2	C51^ii^—C51—H51B	109.1
C38A—C39A—H39A	109.5	C50—C51—H51B	109.1
C38A—C39A—H39B	109.5	H51A—C51—H51B	107.8
H39A—C39A—H39B	109.5		
			
C10—C1—C2—O1	−178.0 (3)	C11—C12—C21—C20	7.2 (5)
C33—C1—C2—O1	−2.3 (5)	C13—C12—C21—C16	2.8 (5)
C10—C1—C2—C3	−0.1 (5)	C11—C12—C21—C16	−170.6 (3)
C33—C1—C2—C3	175.6 (3)	C15—C16—C21—C20	−179.5 (3)
C34—O1—C2—C1	−158.2 (3)	C17—C16—C21—C20	1.0 (5)
C34—O1—C2—C3	23.9 (5)	C15—C16—C21—C12	−1.6 (5)
C1—C2—C3—C4	1.0 (5)	C17—C16—C21—C12	178.9 (3)
O1—C2—C3—C4	178.8 (3)	C18—C17—C22—C23^i^	68.2 (5)
C2—C3—C4—C5	−0.4 (5)	C16—C17—C22—C23^i^	−118.3 (4)
C3—C4—C5—C6	−178.6 (3)	C42—O5—C24—C23	175.9 (3)
C3—C4—C5—C10	−0.9 (5)	C42—O5—C24—C25	−3.6 (5)
C4—C5—C6—C7	174.2 (3)	O5—C24—C23—C32	−178.5 (3)
C10—C5—C6—C7	−3.5 (5)	C25—C24—C23—C32	1.1 (5)
C4—C5—C6—C11	−1.9 (5)	O5—C24—C23—C22^i^	−1.6 (5)
C10—C5—C6—C11	−179.5 (3)	C25—C24—C23—C22^i^	178.0 (3)
C5—C6—C7—O2	−177.0 (3)	C28—C27—C26—C25	−179.8 (3)
C11—C6—C7—O2	−1.0 (5)	C32—C27—C26—C25	−0.4 (5)
C5—C6—C7—C8	3.8 (5)	C27—C26—C25—C24	−1.6 (6)
C11—C6—C7—C8	179.9 (3)	O5—C24—C25—C26	−179.2 (3)
C36—O2—C7—C6	−169.0 (4)	C23—C24—C25—C26	1.3 (5)
C36—O2—C7—C8	10.2 (5)	C44—O6—C29—C28	154.1 (4)
C2—C1—C10—C9	176.8 (3)	C44—O6—C29—C30	−27.5 (6)
C33—C1—C10—C9	1.2 (5)	C31—C30—C29—O6	−176.3 (4)
C2—C1—C10—C5	−1.2 (5)	C31—C30—C29—C28	2.0 (6)
C33—C1—C10—C5	−176.9 (3)	O6—C29—C28—C27	−179.9 (3)
C4—C5—C10—C9	−176.4 (3)	C30—C29—C28—C27	1.7 (5)
C6—C5—C10—C9	1.3 (5)	O6—C29—C28—C33	−3.5 (5)
C4—C5—C10—C1	1.7 (5)	C30—C29—C28—C33	178.1 (3)
C6—C5—C10—C1	179.4 (3)	C26—C27—C28—C29	174.1 (3)
C1—C10—C9—C8	−177.6 (3)	C32—C27—C28—C29	−5.2 (5)
C5—C10—C9—C8	0.5 (5)	C26—C27—C28—C33	−2.2 (5)
C10—C9—C8—C7	−0.2 (5)	C32—C27—C28—C33	178.5 (3)
C6—C7—C8—C9	−2.0 (5)	C13—O3—C40—C41	−176.1 (4)
O2—C7—C8—C9	178.8 (3)	C17—C18—O4—C38B	−127 (2)
C7—C6—C11—C12	113.3 (4)	C19—C18—O4—C38B	56 (2)
C5—C6—C11—C12	−70.7 (4)	C17—C18—O4—C38A	−165.0 (7)
C13—C12—C11—C6	128.5 (4)	C19—C18—O4—C38A	17.6 (8)
C21—C12—C11—C6	−58.1 (5)	C18—O4—C38A—C39A	163.8 (12)
C40—O3—C13—C12	166.1 (4)	C18—O4—C38B—C39B	−143 (3)
C40—O3—C13—C14	−12.6 (6)	C7—O2—C36—C37	176.0 (4)
C21—C12—C13—O3	178.7 (3)	C2—O1—C34—C35	164.4 (4)
C11—C12—C13—O3	−7.6 (5)	C29—C28—C33—C1	121.5 (4)
C21—C12—C13—C14	−2.5 (5)	C27—C28—C33—C1	−62.1 (4)
C11—C12—C13—C14	171.2 (3)	C2—C1—C33—C28	122.4 (3)
C16—C15—C14—C13	0.4 (6)	C10—C1—C33—C28	−62.0 (4)
O3—C13—C14—C15	179.6 (4)	C24—C23—C32—C31	174.0 (3)
C12—C13—C14—C15	0.9 (6)	C22^i^—C23—C32—C31	−2.8 (5)
C14—C15—C16—C17	179.5 (4)	C24—C23—C32—C27	−3.2 (5)
C14—C15—C16—C21	−0.1 (6)	C22^i^—C23—C32—C27	−179.9 (3)
C15—C16—C17—C18	−178.8 (3)	C28—C27—C32—C23	−177.9 (3)
C21—C16—C17—C18	0.7 (5)	C26—C27—C32—C23	2.8 (5)
C15—C16—C17—C22	7.6 (5)	C28—C27—C32—C31	4.8 (5)
C21—C16—C17—C22	−172.8 (3)	C26—C27—C32—C31	−174.5 (3)
C16—C17—C18—O4	−178.2 (3)	C29—C30—C31—C32	−2.3 (6)
C22—C17—C18—O4	−4.5 (5)	C23—C32—C31—C30	−178.3 (3)
C16—C17—C18—C19	−0.8 (5)	C27—C32—C31—C30	−1.0 (5)
C22—C17—C18—C19	172.9 (3)	C24—O5—C42—C43	−177.2 (3)
C21—C20—C19—C18	2.6 (6)	C29—O6—C44—C45	−172.2 (4)
O4—C18—C19—C20	176.5 (4)	Cl1—C46—C47—C48	57.0 (9)
C17—C18—C19—C20	−0.8 (6)	C46—C47—C48—C48^i^	61.7 (7)
C19—C20—C21—C12	179.5 (4)	Cl2—C49—C50—C51	−179.3 (3)
C19—C20—C21—C16	−2.6 (6)	C49—C50—C51—C51^ii^	179.4 (4)
C13—C12—C21—C20	−179.4 (3)		

Symmetry codes: (i) −*x*+1, *y*, −*z*+1/2; (ii) −*x*+3/2, −*y*+1/2, −*z*+1.

### Perethoxy-prism[6]arene 1,6-diiodohexane disolvate (PS6_HexI2) Crystal data

**Table:**

C_90_H_96_O_12_·2C_6_H_12_I_2_	*F*(000) = 4160
*M**_r_* = 2045.57	*D*_x_ = 1.447 Mg m^−^^3^
Monoclinic, *C*2/*c*	Mo *K*α radiation, λ = 0.71075 Å
*a* = 38.728 (3) Å	Cell parameters from 15264 reflections
*b* = 10.5427 (6) Å	θ = 3.2–25.0°
*c* = 24.0234 (16) Å	µ = 1.39 mm^−^^1^
β = 106.814 (8)°	*T* = 293 K
*V* = 9389.3 (11) Å^3^	Block, colorless
*Z* = 4	0.20 × 0.17 × 0.15 mm

### Perethoxy-prism[6]arene 1,6-diiodohexane disolvate (PS6_HexI2) Data collection

**Table:**

Rigaku R-AXIS RAPID diffractometer	4349 reflections with *I* > 2σ(*I*)
Detector resolution: 10.000 pixels mm^-1^	*R*_int_ = 0.077
ω scans	θ_max_ = 25.0°, θ_min_ = 3.2°
Absorption correction: multi-scan (ABSCOR; Higashi, 1995)	*h* = −44→46
*T*_min_ = 0.452, *T*_max_ = 0.884	*k* = −12→12
27851 measured reflections	*l* = −28→28
8193 independent reflections	

### Perethoxy-prism[6]arene 1,6-diiodohexane disolvate (PS6_HexI2) Refinement

**Table:**

Refinement on *F*^2^	Primary atom site location: structure-invariant direct methods
Least-squares matrix: full	Hydrogen site location: inferred from neighbouring sites
*R*[*F*^2^ > 2σ(*F*^2^)] = 0.069	H-atom parameters constrained
*wR*(*F*^2^) = 0.192	*w* = 1/[σ^2^(*F*_o_^2^) + (0.0908*P*)^2^ + 16.5419*P*] where *P* = (*F*_o_^2^ + 2*F*_c_^2^)/3
*S* = 1.02	(Δ/σ)_max_ = 0.001
8193 reflections	Δρ_max_ = 0.70 e Å^−^^3^
532 parameters	Δρ_min_ = −0.77 e Å^−^^3^
46 restraints	

### Perethoxy-prism[6]arene 1,6-diiodohexane disolvate (PS6_HexI2) Special details

**Table:**

Geometry. All esds (except the esd in the dihedral angle between two l.s. planes) are estimated using the full covariance matrix. The cell esds are taken into account individually in the estimation of esds in distances, angles and torsion angles; correlations between esds in cell parameters are only used when they are defined by crystal symmetry. An approximate (isotropic) treatment of cell esds is used for estimating esds involving l.s. planes.

### Perethoxy-prism[6]arene 1,6-diiodohexane disolvate (PS6_HexI2) Fractional atomic coordinates and isotropic or equivalent isotropic displacement parameters (Å^2^)

**Table:**

	*x*	*y*	*z*	*U*_iso_*/*U*_eq_	
I1	0.56133 (2)	1.12590 (7)	0.29126 (3)	0.0893 (3)	
O1	0.68726 (12)	0.9155 (5)	0.2043 (2)	0.0639 (14)	
C1	0.66315 (16)	0.6730 (6)	0.3135 (3)	0.0377 (15)	
C4	0.64994 (17)	0.6201 (6)	0.4197 (3)	0.0432 (16)	
H4	0.645166	0.600207	0.454496	0.052*	
O4	0.65571 (12)	1.0642 (4)	0.46814 (19)	0.0494 (11)	
C5	0.65374 (15)	0.7480 (5)	0.4062 (3)	0.0350 (14)	
O5	0.64596 (12)	0.5905 (5)	0.5595 (2)	0.0595 (13)	
C6	0.65149 (15)	0.8480 (5)	0.4446 (3)	0.0355 (15)	
O6	0.47670 (11)	0.9847 (4)	0.40813 (19)	0.0506 (11)	
C7	0.65716 (15)	0.9700 (6)	0.4293 (3)	0.0366 (14)	
C8	0.66516 (17)	0.9956 (6)	0.3773 (3)	0.0434 (16)	
H8	0.669578	1.078700	0.368333	0.052*	
C9	0.66660 (16)	0.9010 (6)	0.3395 (3)	0.0390 (15)	
H9	0.671337	0.920996	0.304671	0.047*	
C51	0.7525 (2)	0.8183 (8)	0.4926 (4)	0.080 (2)	
H51A	0.734746	0.839372	0.456215	0.096*	
H51B	0.776216	0.827867	0.487069	0.096*	
C50	0.7490 (2)	0.9106 (8)	0.5388 (4)	0.082 (2)	
H50A	0.725478	0.900458	0.544926	0.098*	
H50B	0.767105	0.890904	0.575155	0.098*	
C3	0.65303 (18)	0.5244 (6)	0.3835 (3)	0.0467 (17)	
H3	0.650445	0.440814	0.393951	0.056*	
O3	0.49439 (13)	0.5548 (5)	0.3944 (2)	0.0629 (13)	
C2	0.66008 (17)	0.5502 (6)	0.3305 (3)	0.0416 (15)	
I2	0.74942 (2)	1.18026 (7)	0.58573 (4)	0.1109 (3)	
O2	0.66407 (12)	0.4562 (4)	0.29368 (19)	0.0505 (12)	
C12	0.61111 (16)	0.7585 (6)	0.5057 (3)	0.0361 (14)	
C11	0.64584 (16)	0.8221 (6)	0.5036 (3)	0.0377 (14)	
H11A	0.665739	0.769788	0.525533	0.045*	
H11B	0.647612	0.902444	0.523862	0.045*	
C10	0.66105 (15)	0.7741 (6)	0.3520 (2)	0.0341 (14)	
C14	0.58096 (19)	0.5845 (6)	0.5379 (3)	0.0492 (17)	
H14	0.582407	0.508483	0.558060	0.059*	
C13	0.61251 (17)	0.6438 (6)	0.5351 (3)	0.0433 (16)	
C15	0.54843 (18)	0.6352 (6)	0.5120 (3)	0.0461 (16)	
H15	0.527919	0.591971	0.514124	0.055*	
C17	0.51062 (17)	0.8104 (6)	0.4553 (3)	0.0412 (15)	
C16	0.54417 (16)	0.7520 (6)	0.4814 (3)	0.0378 (15)	
C22	0.47517 (16)	0.7504 (7)	0.4547 (3)	0.0441 (16)	
H22A	0.462031	0.810364	0.471469	0.053*	
H22B	0.480318	0.676923	0.480069	0.053*	
C21	0.57746 (16)	0.8140 (6)	0.4802 (2)	0.0355 (14)	
C20	0.57429 (16)	0.9373 (6)	0.4551 (2)	0.0387 (15)	
H20	0.595136	0.980109	0.454276	0.046*	
C19	0.54171 (17)	0.9947 (6)	0.4322 (3)	0.0424 (15)	
H19	0.540712	1.076104	0.416913	0.051*	
C18	0.50957 (16)	0.9314 (6)	0.4315 (3)	0.0392 (15)	
C24	0.46082 (17)	0.6063 (6)	0.3684 (3)	0.0452 (16)	
C23	0.45027 (16)	0.7080 (6)	0.3959 (3)	0.0414 (15)	
C27	0.60851 (16)	0.7086 (6)	0.1808 (3)	0.0389 (15)	
C26	0.59565 (18)	0.6043 (6)	0.2051 (3)	0.0474 (16)	
H26	0.610700	0.567616	0.238564	0.057*	
C25	0.56240 (18)	0.5545 (6)	0.1821 (3)	0.0533 (18)	
H25	0.554985	0.485669	0.199943	0.064*	
C30	0.63019 (18)	0.9153 (7)	0.1283 (3)	0.0531 (18)	
H30	0.637265	0.985529	0.110921	0.064*	
C29	0.65376 (17)	0.8672 (6)	0.1789 (3)	0.0464 (17)	
C28	0.64354 (16)	0.7607 (6)	0.2060 (3)	0.0389 (15)	
C34	0.6522 (2)	0.3342 (7)	0.2997 (3)	0.067 (2)	
H34A	0.627115	0.336511	0.299598	0.080*	
H34B	0.666312	0.298299	0.336365	0.080*	
C33	0.67158 (17)	0.6974 (6)	0.2566 (3)	0.0400 (15)	
H33A	0.677646	0.616446	0.242728	0.048*	
H33B	0.693189	0.749212	0.265380	0.048*	
C32	0.58404 (16)	0.7601 (6)	0.1283 (2)	0.0369 (14)	
C31	0.59773 (18)	0.8648 (6)	0.1037 (3)	0.0495 (17)	
H31	0.583479	0.899302	0.069052	0.059*	
C40	0.47465 (18)	1.1141 (6)	0.3889 (3)	0.0514 (17)	
H40A	0.484876	1.121769	0.356627	0.062*	
H40B	0.488366	1.167970	0.420239	0.062*	
C39	0.6919 (2)	0.4877 (11)	0.6297 (4)	0.106 (4)	
H39A	0.696510	0.431617	0.662493	0.159*	
H39B	0.700599	0.450062	0.599956	0.159*	
H39C	0.703933	0.566984	0.641498	0.159*	
C38	0.6522 (2)	0.5101 (9)	0.6064 (3)	0.075 (2)	
H38A	0.639816	0.430254	0.594661	0.089*	
H38B	0.643160	0.547550	0.636336	0.089*	
C37	0.6393 (3)	1.2653 (7)	0.4923 (4)	0.079 (3)	
H37A	0.635691	1.352246	0.480115	0.118*	
H37B	0.617099	1.231123	0.496139	0.118*	
H37C	0.657543	1.260496	0.529035	0.118*	
C36	0.6507 (2)	1.1922 (6)	0.4489 (3)	0.0521 (18)	
H36A	0.632396	1.196804	0.411572	0.062*	
H36B	0.672993	1.226287	0.444593	0.062*	
C35	0.6559 (3)	0.2548 (8)	0.2510 (4)	0.093 (3)	
H35A	0.647661	0.170337	0.255099	0.139*	
H35B	0.641662	0.290255	0.214843	0.139*	
H35C	0.680752	0.252148	0.251509	0.139*	
C42	0.5097 (2)	0.4753 (11)	0.3589 (5)	0.114 (4)	
H42A	0.507684	0.517106	0.322153	0.137*	
H42B	0.496467	0.395986	0.350856	0.137*	
C41	0.43599 (19)	1.1554 (7)	0.3701 (3)	0.061 (2)	
H41A	0.434534	1.241933	0.357194	0.092*	
H41B	0.422582	1.102313	0.338820	0.092*	
H41C	0.426063	1.148385	0.402255	0.092*	
C43	0.5483 (3)	0.4491 (14)	0.3894 (6)	0.157 (6)	
H43A	0.558290	0.396076	0.365400	0.236*	
H43B	0.561426	0.527635	0.396890	0.236*	
H43C	0.550233	0.406771	0.425532	0.236*	
C45	0.7358 (3)	1.0574 (11)	0.2155 (5)	0.118 (4)	
H45A	0.744594	1.128269	0.198495	0.177*	
H45B	0.734272	1.080433	0.253380	0.177*	
H45C	0.752081	0.987119	0.218952	0.177*	
C44	0.6990 (2)	1.0202 (8)	0.1774 (3)	0.069 (2)	
H44A	0.682302	1.090484	0.173563	0.083*	
H44B	0.700191	0.996753	0.138981	0.083*	
C46	0.5677 (2)	0.9358 (10)	0.2635 (5)	0.097 (3)	
H46A	0.557385	0.931225	0.221638	0.116*	
H46B	0.593289	0.917814	0.272127	0.116*	
C49	0.7535 (2)	1.0452 (8)	0.5228 (5)	0.093 (3)	
H49A	0.735317	1.064157	0.486455	0.112*	
H49B	0.776919	1.054199	0.516084	0.112*	
C48	0.5108 (2)	0.8462 (8)	0.2812 (3)	0.074 (2)	
H48A	0.503209	0.775195	0.300553	0.088*	
H48B	0.505687	0.923420	0.299276	0.088*	
C47	0.5511 (3)	0.8370 (9)	0.2904 (4)	0.094 (3)	
H47A	0.562902	0.837729	0.331884	0.112*	
H47B	0.556070	0.755456	0.275659	0.112*	

### Perethoxy-prism[6]arene 1,6-diiodohexane disolvate (PS6_HexI2) Atomic displacement parameters (Å^2^)

**Table:**

	*U*^11^	*U*^22^	*U*^33^	*U*^12^	*U*^13^	*U*^23^
I1	0.0947 (5)	0.1068 (5)	0.0699 (4)	−0.0250 (4)	0.0291 (3)	−0.0089 (4)
O1	0.048 (3)	0.075 (3)	0.062 (3)	−0.015 (2)	0.005 (2)	0.025 (3)
C1	0.040 (4)	0.040 (4)	0.032 (3)	0.004 (3)	0.010 (3)	0.008 (3)
C4	0.061 (4)	0.036 (4)	0.037 (4)	0.004 (3)	0.022 (3)	0.008 (3)
O4	0.073 (3)	0.036 (3)	0.045 (3)	−0.005 (2)	0.027 (2)	−0.001 (2)
C5	0.038 (4)	0.031 (4)	0.034 (3)	0.004 (3)	0.009 (3)	0.001 (3)
O5	0.055 (3)	0.063 (3)	0.064 (3)	0.011 (2)	0.023 (2)	0.037 (3)
C6	0.031 (3)	0.038 (4)	0.038 (4)	−0.001 (3)	0.010 (3)	0.007 (3)
O6	0.047 (3)	0.049 (3)	0.053 (3)	0.000 (2)	0.011 (2)	0.005 (2)
C7	0.040 (4)	0.033 (4)	0.036 (4)	0.000 (3)	0.010 (3)	−0.003 (3)
C8	0.061 (4)	0.026 (3)	0.047 (4)	−0.004 (3)	0.022 (3)	0.009 (3)
C9	0.051 (4)	0.037 (4)	0.031 (3)	−0.004 (3)	0.015 (3)	0.004 (3)
C51	0.059 (5)	0.087 (5)	0.088 (6)	0.003 (5)	0.011 (5)	0.020 (5)
C50	0.068 (5)	0.080 (5)	0.089 (6)	−0.002 (4)	0.011 (5)	0.005 (5)
C3	0.062 (4)	0.029 (4)	0.051 (4)	0.006 (3)	0.020 (3)	0.015 (3)
O3	0.054 (3)	0.061 (3)	0.067 (3)	0.005 (2)	0.007 (3)	−0.012 (3)
C2	0.052 (4)	0.032 (4)	0.040 (4)	0.012 (3)	0.013 (3)	0.001 (3)
I2	0.1102 (6)	0.0870 (5)	0.1344 (7)	−0.0032 (4)	0.0338 (5)	0.0104 (5)
O2	0.075 (3)	0.031 (3)	0.050 (3)	0.007 (2)	0.026 (2)	0.002 (2)
C12	0.044 (4)	0.034 (3)	0.034 (3)	−0.004 (3)	0.017 (3)	−0.002 (3)
C11	0.046 (4)	0.037 (4)	0.035 (3)	0.000 (3)	0.019 (3)	0.004 (3)
C10	0.034 (3)	0.036 (3)	0.031 (3)	−0.003 (3)	0.008 (3)	0.005 (3)
C14	0.060 (5)	0.040 (4)	0.047 (4)	−0.004 (3)	0.013 (3)	0.012 (3)
C13	0.049 (4)	0.046 (4)	0.038 (4)	0.003 (3)	0.018 (3)	0.010 (3)
C15	0.047 (4)	0.046 (4)	0.047 (4)	−0.010 (3)	0.015 (3)	0.009 (4)
C17	0.050 (4)	0.047 (4)	0.031 (3)	−0.014 (3)	0.018 (3)	−0.004 (3)
C16	0.043 (4)	0.038 (4)	0.031 (3)	−0.003 (3)	0.009 (3)	−0.003 (3)
C22	0.045 (4)	0.052 (4)	0.037 (4)	−0.006 (3)	0.015 (3)	0.000 (3)
C21	0.043 (4)	0.039 (4)	0.025 (3)	−0.002 (3)	0.010 (3)	0.001 (3)
C20	0.040 (4)	0.044 (4)	0.031 (3)	−0.007 (3)	0.010 (3)	−0.001 (3)
C19	0.054 (4)	0.034 (3)	0.039 (4)	0.005 (3)	0.014 (3)	0.010 (3)
C18	0.036 (4)	0.049 (4)	0.031 (3)	0.000 (3)	0.006 (3)	−0.002 (3)
C24	0.046 (4)	0.045 (4)	0.044 (4)	0.002 (3)	0.013 (3)	−0.003 (3)
C23	0.040 (4)	0.044 (4)	0.041 (4)	−0.008 (3)	0.013 (3)	0.002 (3)
C27	0.049 (4)	0.039 (4)	0.035 (4)	0.006 (3)	0.021 (3)	0.008 (3)
C26	0.051 (4)	0.047 (4)	0.041 (4)	0.001 (3)	0.008 (3)	0.003 (3)
C25	0.056 (5)	0.046 (4)	0.059 (5)	0.001 (3)	0.018 (4)	0.014 (4)
C30	0.047 (4)	0.061 (5)	0.052 (4)	−0.006 (3)	0.017 (4)	0.024 (4)
C29	0.042 (4)	0.058 (4)	0.042 (4)	−0.003 (3)	0.017 (3)	0.007 (4)
C28	0.041 (4)	0.047 (4)	0.033 (3)	0.008 (3)	0.017 (3)	0.006 (3)
C34	0.102 (6)	0.046 (5)	0.058 (5)	0.001 (4)	0.034 (5)	0.003 (4)
C33	0.047 (4)	0.044 (4)	0.033 (3)	0.009 (3)	0.017 (3)	0.007 (3)
C32	0.045 (4)	0.039 (4)	0.029 (3)	0.007 (3)	0.015 (3)	−0.001 (3)
C31	0.045 (4)	0.059 (4)	0.044 (4)	0.003 (3)	0.013 (3)	0.020 (4)
C40	0.061 (5)	0.048 (4)	0.041 (4)	0.004 (3)	0.008 (3)	−0.003 (3)
C39	0.071 (6)	0.150 (9)	0.092 (7)	0.035 (6)	0.015 (5)	0.065 (7)
C38	0.087 (6)	0.099 (7)	0.044 (4)	0.022 (5)	0.028 (4)	0.029 (5)
C37	0.131 (8)	0.041 (4)	0.078 (6)	0.014 (5)	0.053 (6)	0.005 (4)
C36	0.069 (5)	0.033 (4)	0.057 (5)	−0.002 (3)	0.023 (4)	0.001 (3)
C35	0.155 (9)	0.057 (5)	0.076 (6)	−0.011 (5)	0.050 (6)	−0.014 (5)
C42	0.089 (7)	0.112 (8)	0.123 (9)	0.045 (6)	0.003 (6)	−0.032 (7)
C41	0.055 (5)	0.069 (5)	0.060 (5)	0.016 (4)	0.017 (4)	−0.001 (4)
C43	0.103 (8)	0.196 (14)	0.153 (12)	0.069 (9)	0.005 (7)	−0.071 (10)
C45	0.078 (7)	0.130 (9)	0.128 (9)	−0.044 (6)	0.000 (6)	0.020 (8)
C44	0.068 (5)	0.074 (5)	0.065 (5)	−0.015 (4)	0.020 (4)	0.014 (4)
C46	0.074 (6)	0.116 (8)	0.105 (8)	0.014 (5)	0.035 (5)	−0.009 (6)
C49	0.058 (5)	0.088 (5)	0.121 (8)	−0.003 (5)	0.006 (5)	0.027 (6)
C48	0.109 (6)	0.062 (5)	0.054 (4)	−0.003 (4)	0.030 (5)	−0.001 (4)
C47	0.106 (6)	0.090 (7)	0.081 (7)	0.027 (5)	0.021 (6)	−0.008 (5)

### Perethoxy-prism[6]arene 1,6-diiodohexane disolvate (PS6_HexI2) Geometric parameters (Å, º)

**Table:**

I1—C46	2.149 (10)	C27—C26	1.402 (9)
O1—C29	1.363 (8)	C27—C28	1.426 (8)
O1—C44	1.418 (8)	C27—C32	1.446 (8)
C1—C2	1.373 (8)	C26—C25	1.352 (9)
C1—C10	1.428 (8)	C26—H26	0.9300
C1—C33	1.516 (8)	C25—H25	0.9300
C4—C3	1.360 (9)	C30—C31	1.336 (9)
C4—C5	1.406 (8)	C30—C29	1.387 (9)
C4—H4	0.9300	C30—H30	0.9300
O4—C7	1.375 (7)	C29—C28	1.410 (9)
O4—C36	1.421 (7)	C28—C33	1.529 (8)
C5—C6	1.421 (8)	C34—C35	1.479 (11)
C5—C10	1.436 (8)	C34—H34A	0.9700
O5—C38	1.375 (8)	C34—H34B	0.9700
O5—C13	1.378 (8)	C33—H33A	0.9700
C6—C7	1.373 (8)	C33—H33B	0.9700
C6—C11	1.519 (8)	C32—C31	1.425 (9)
O6—C18	1.355 (7)	C31—H31	0.9300
O6—C40	1.435 (8)	C40—C41	1.498 (9)
C7—C8	1.398 (8)	C40—H40A	0.9700
C8—C9	1.360 (8)	C40—H40B	0.9700
C8—H8	0.9300	C39—C38	1.492 (11)
C9—C10	1.402 (8)	C39—H39A	0.9600
C9—H9	0.9300	C39—H39B	0.9600
C51—C51^i^	1.509 (16)	C39—H39C	0.9600
C51—C50	1.511 (13)	C38—H38A	0.9700
C51—H51A	0.9700	C38—H38B	0.9700
C51—H51B	0.9700	C37—C36	1.462 (10)
C50—C49	1.495 (12)	C37—H37A	0.9600
C50—H50A	0.9700	C37—H37B	0.9600
C50—H50B	0.9700	C37—H37C	0.9600
C3—C2	1.403 (9)	C36—H36A	0.9700
C3—H3	0.9300	C36—H36B	0.9700
O3—C24	1.380 (8)	C35—H35A	0.9600
O3—C42	1.440 (10)	C35—H35B	0.9600
C2—O2	1.367 (7)	C35—H35C	0.9600
I2—C49	2.116 (11)	C42—C43	1.490 (8)
O2—C34	1.388 (8)	C42—H42A	0.9700
C12—C13	1.394 (9)	C42—H42B	0.9700
C12—C21	1.398 (8)	C41—H41A	0.9600
C12—C11	1.517 (8)	C41—H41B	0.9600
C11—H11A	0.9700	C41—H41C	0.9600
C11—H11B	0.9700	C43—H43A	0.9600
C14—C15	1.344 (9)	C43—H43B	0.9600
C14—C13	1.391 (9)	C43—H43C	0.9600
C14—H14	0.9300	C45—C44	1.508 (11)
C15—C16	1.418 (9)	C45—H45A	0.9600
C15—H15	0.9300	C45—H45B	0.9600
C17—C18	1.394 (9)	C45—H45C	0.9600
C17—C16	1.411 (9)	C44—H44A	0.9700
C17—C22	1.508 (8)	C44—H44B	0.9700
C16—C21	1.453 (8)	C46—C47	1.469 (13)
C22—C23	1.528 (9)	C46—H46A	0.9700
C22—H22A	0.9700	C46—H46B	0.9700
C22—H22B	0.9700	C49—H49A	0.9700
C21—C20	1.423 (9)	C49—H49B	0.9700
C20—C19	1.364 (8)	C48—C48^ii^	1.490 (16)
C20—H20	0.9300	C48—C47	1.514 (12)
C19—C18	1.408 (8)	C48—H48A	0.9700
C19—H19	0.9300	C48—H48B	0.9700
C24—C23	1.382 (9)	C47—H47A	0.9700
C24—C25^ii^	1.395 (9)	C47—H47B	0.9700
C23—C32^ii^	1.399 (8)		
			
C29—O1—C44	118.4 (5)	C29—C28—C27	118.1 (5)
C2—C1—C10	119.0 (5)	C29—C28—C33	119.0 (5)
C2—C1—C33	119.2 (6)	C27—C28—C33	122.6 (5)
C10—C1—C33	121.6 (5)	O2—C34—C35	109.4 (6)
C3—C4—C5	121.9 (6)	O2—C34—H34A	109.8
C3—C4—H4	119.0	C35—C34—H34A	109.8
C5—C4—H4	119.0	O2—C34—H34B	109.8
C7—O4—C36	119.6 (5)	C35—C34—H34B	109.8
C4—C5—C6	122.1 (5)	H34A—C34—H34B	108.2
C4—C5—C10	117.0 (5)	C1—C33—C28	120.2 (5)
C6—C5—C10	120.9 (5)	C1—C33—H33A	107.3
C38—O5—C13	121.4 (5)	C28—C33—H33A	107.3
C7—C6—C5	118.4 (5)	C1—C33—H33B	107.3
C7—C6—C11	119.7 (5)	C28—C33—H33B	107.3
C5—C6—C11	121.7 (5)	H33A—C33—H33B	106.9
C18—O6—C40	119.0 (5)	C23^ii^—C32—C31	123.6 (6)
C6—C7—O4	117.0 (5)	C23^ii^—C32—C27	121.3 (5)
C6—C7—C8	120.9 (6)	C31—C32—C27	115.1 (6)
O4—C7—C8	122.1 (5)	C30—C31—C32	122.7 (6)
C9—C8—C7	121.2 (6)	C30—C31—H31	118.6
C9—C8—H8	119.4	C32—C31—H31	118.6
C7—C8—H8	119.4	O6—C40—C41	109.3 (6)
C8—C9—C10	121.2 (5)	O6—C40—H40A	109.8
C8—C9—H9	119.4	C41—C40—H40A	109.8
C10—C9—H9	119.4	O6—C40—H40B	109.8
C51^i^—C51—C50	113.4 (10)	C41—C40—H40B	109.8
C51^i^—C51—H51A	108.9	H40A—C40—H40B	108.3
C50—C51—H51A	108.9	C38—C39—H39A	109.5
C51^i^—C51—H51B	108.9	C38—C39—H39B	109.5
C50—C51—H51B	108.9	H39A—C39—H39B	109.5
H51A—C51—H51B	107.7	C38—C39—H39C	109.5
C49—C50—C51	112.3 (8)	H39A—C39—H39C	109.5
C49—C50—H50A	109.1	H39B—C39—H39C	109.5
C51—C50—H50A	109.1	O5—C38—C39	108.8 (7)
C49—C50—H50B	109.1	O5—C38—H38A	109.9
C51—C50—H50B	109.1	C39—C38—H38A	109.9
H50A—C50—H50B	107.9	O5—C38—H38B	109.9
C4—C3—C2	120.9 (6)	C39—C38—H38B	109.9
C4—C3—H3	119.6	H38A—C38—H38B	108.3
C2—C3—H3	119.6	C36—C37—H37A	109.5
C24—O3—C42	117.1 (6)	C36—C37—H37B	109.5
O2—C2—C1	117.1 (5)	H37A—C37—H37B	109.5
O2—C2—C3	122.3 (5)	C36—C37—H37C	109.5
C1—C2—C3	120.6 (6)	H37A—C37—H37C	109.5
C2—O2—C34	120.1 (5)	H37B—C37—H37C	109.5
C13—C12—C21	118.7 (5)	O4—C36—C37	108.1 (5)
C13—C12—C11	119.7 (5)	O4—C36—H36A	110.1
C21—C12—C11	121.6 (5)	C37—C36—H36A	110.1
C12—C11—C6	118.6 (5)	O4—C36—H36B	110.1
C12—C11—H11A	107.7	C37—C36—H36B	110.1
C6—C11—H11A	107.7	H36A—C36—H36B	108.4
C12—C11—H11B	107.7	C34—C35—H35A	109.5
C6—C11—H11B	107.7	C34—C35—H35B	109.5
H11A—C11—H11B	107.1	H35A—C35—H35B	109.5
C9—C10—C1	122.1 (5)	C34—C35—H35C	109.5
C9—C10—C5	117.4 (5)	H35A—C35—H35C	109.5
C1—C10—C5	120.5 (5)	H35B—C35—H35C	109.5
C15—C14—C13	121.2 (6)	O3—C42—C43	110.1 (8)
C15—C14—H14	119.4	O3—C42—H42A	109.6
C13—C14—H14	119.4	C43—C42—H42A	109.6
O5—C13—C14	121.8 (6)	O3—C42—H42B	109.6
O5—C13—C12	117.6 (5)	C43—C42—H42B	109.6
C14—C13—C12	120.6 (6)	H42A—C42—H42B	108.2
C14—C15—C16	122.5 (6)	C40—C41—H41A	109.5
C14—C15—H15	118.7	C40—C41—H41B	109.5
C16—C15—H15	118.7	H41A—C41—H41B	109.5
C18—C17—C16	119.7 (5)	C40—C41—H41C	109.5
C18—C17—C22	117.5 (6)	H41A—C41—H41C	109.5
C16—C17—C22	122.7 (6)	H41B—C41—H41C	109.5
C17—C16—C15	124.3 (6)	C42—C43—H43A	109.5
C17—C16—C21	120.2 (6)	C42—C43—H43B	109.5
C15—C16—C21	115.4 (5)	H43A—C43—H43B	109.5
C17—C22—C23	117.4 (5)	C42—C43—H43C	109.5
C17—C22—H22A	107.9	H43A—C43—H43C	109.5
C23—C22—H22A	107.9	H43B—C43—H43C	109.5
C17—C22—H22B	107.9	C44—C45—H45A	109.5
C23—C22—H22B	107.9	C44—C45—H45B	109.5
H22A—C22—H22B	107.2	H45A—C45—H45B	109.5
C12—C21—C20	121.5 (5)	C44—C45—H45C	109.5
C12—C21—C16	121.6 (6)	H45A—C45—H45C	109.5
C20—C21—C16	116.8 (5)	H45B—C45—H45C	109.5
C19—C20—C21	122.2 (6)	O1—C44—C45	107.3 (7)
C19—C20—H20	118.9	O1—C44—H44A	110.3
C21—C20—H20	118.9	C45—C44—H44A	110.3
C20—C19—C18	120.4 (6)	O1—C44—H44B	110.3
C20—C19—H19	119.8	C45—C44—H44B	110.3
C18—C19—H19	119.8	H44A—C44—H44B	108.5
O6—C18—C17	117.5 (5)	C47—C46—I1	114.9 (7)
O6—C18—C19	122.0 (6)	C47—C46—H46A	108.6
C17—C18—C19	120.5 (6)	I1—C46—H46A	108.6
O3—C24—C23	117.3 (6)	C47—C46—H46B	108.6
O3—C24—C25^ii^	121.7 (6)	I1—C46—H46B	108.6
C23—C24—C25^ii^	120.9 (6)	H46A—C46—H46B	107.5
C24—C23—C32^ii^	118.8 (6)	C50—C49—I2	114.6 (7)
C24—C23—C22	118.3 (6)	C50—C49—H49A	108.6
C32^ii^—C23—C22	122.7 (6)	I2—C49—H49A	108.6
C26—C27—C28	122.3 (6)	C50—C49—H49B	108.6
C26—C27—C32	115.8 (6)	I2—C49—H49B	108.6
C28—C27—C32	121.9 (5)	H49A—C49—H49B	107.6
C25—C26—C27	123.0 (6)	C48^ii^—C48—C47	113.6 (9)
C25—C26—H26	118.5	C48^ii^—C48—H48A	108.8
C27—C26—H26	118.5	C47—C48—H48A	108.8
C26—C25—C24^ii^	120.1 (6)	C48^ii^—C48—H48B	108.8
C26—C25—H25	119.9	C47—C48—H48B	108.8
C24^ii^—C25—H25	119.9	H48A—C48—H48B	107.7
C31—C30—C29	122.6 (6)	C46—C47—C48	117.1 (8)
C31—C30—H30	118.7	C46—C47—H47A	108.0
C29—C30—H30	118.7	C48—C47—H47A	108.0
O1—C29—C30	123.7 (6)	C46—C47—H47B	108.0
O1—C29—C28	116.7 (6)	C48—C47—H47B	108.0
C30—C29—C28	119.6 (6)	H47A—C47—H47B	107.3
			
C3—C4—C5—C6	−178.2 (6)	C11—C12—C21—C16	178.9 (5)
C3—C4—C5—C10	0.1 (9)	C17—C16—C21—C12	−179.9 (6)
C4—C5—C6—C7	177.0 (6)	C15—C16—C21—C12	2.4 (8)
C10—C5—C6—C7	−1.2 (8)	C17—C16—C21—C20	3.9 (8)
C4—C5—C6—C11	1.7 (9)	C15—C16—C21—C20	−173.8 (5)
C10—C5—C6—C11	−176.6 (5)	C12—C21—C20—C19	−177.3 (6)
C5—C6—C7—O4	−178.4 (5)	C16—C21—C20—C19	−1.1 (8)
C11—C6—C7—O4	−2.9 (8)	C21—C20—C19—C18	−1.4 (9)
C5—C6—C7—C8	−0.4 (9)	C40—O6—C18—C17	173.5 (5)
C11—C6—C7—C8	175.1 (5)	C40—O6—C18—C19	−6.5 (8)
C36—O4—C7—C6	−161.5 (5)	C16—C17—C18—O6	−178.5 (5)
C36—O4—C7—C8	20.5 (8)	C22—C17—C18—O6	−1.9 (8)
C6—C7—C8—C9	1.9 (9)	C16—C17—C18—C19	1.6 (9)
O4—C7—C8—C9	179.8 (6)	C22—C17—C18—C19	178.1 (5)
C7—C8—C9—C10	−1.7 (9)	C20—C19—C18—O6	−178.7 (6)
C51^i^—C51—C50—C49	−179.0 (9)	C20—C19—C18—C17	1.2 (9)
C5—C4—C3—C2	−0.2 (10)	C42—O3—C24—C23	−162.7 (7)
C10—C1—C2—O2	−176.8 (5)	C42—O3—C24—C25^ii^	20.4 (10)
C33—C1—C2—O2	−1.5 (8)	O3—C24—C23—C32^ii^	−178.1 (5)
C10—C1—C2—C3	3.4 (9)	C25^ii^—C24—C23—C32^ii^	−1.1 (9)
C33—C1—C2—C3	178.7 (6)	O3—C24—C23—C22	−4.0 (9)
C4—C3—C2—O2	178.6 (6)	C25^ii^—C24—C23—C22	173.0 (6)
C4—C3—C2—C1	−1.6 (10)	C17—C22—C23—C24	68.8 (8)
C1—C2—O2—C34	−162.9 (6)	C17—C22—C23—C32^ii^	−117.3 (7)
C3—C2—O2—C34	16.9 (9)	C28—C27—C26—C25	179.4 (6)
C13—C12—C11—C6	121.2 (6)	C32—C27—C26—C25	−0.9 (9)
C21—C12—C11—C6	−60.6 (8)	C27—C26—C25—C24^ii^	0.6 (11)
C7—C6—C11—C12	121.4 (6)	C44—O1—C29—C30	−0.7 (10)
C5—C6—C11—C12	−63.3 (8)	C44—O1—C29—C28	178.0 (6)
C8—C9—C10—C1	−178.7 (6)	C31—C30—C29—O1	178.7 (7)
C8—C9—C10—C5	0.1 (8)	C31—C30—C29—C28	0.1 (11)
C2—C1—C10—C9	175.2 (6)	O1—C29—C28—C27	179.3 (6)
C33—C1—C10—C9	0.1 (9)	C30—C29—C28—C27	−1.9 (9)
C2—C1—C10—C5	−3.6 (8)	O1—C29—C28—C33	−7.1 (9)
C33—C1—C10—C5	−178.8 (5)	C30—C29—C28—C33	171.6 (6)
C4—C5—C10—C9	−177.0 (5)	C26—C27—C28—C29	−178.8 (6)
C6—C5—C10—C9	1.3 (8)	C32—C27—C28—C29	1.5 (9)
C4—C5—C10—C1	1.9 (8)	C26—C27—C28—C33	7.9 (9)
C6—C5—C10—C1	−179.8 (5)	C32—C27—C28—C33	−171.8 (5)
C38—O5—C13—C14	−27.6 (10)	C2—O2—C34—C35	173.8 (7)
C38—O5—C13—C12	154.1 (7)	C2—C1—C33—C28	113.4 (7)
C15—C14—C13—O5	−177.4 (6)	C10—C1—C33—C28	−71.5 (8)
C15—C14—C13—C12	0.8 (10)	C29—C28—C33—C1	128.3 (6)
C21—C12—C13—O5	179.6 (5)	C27—C28—C33—C1	−58.4 (8)
C11—C12—C13—O5	−2.2 (9)	C26—C27—C32—C23^ii^	0.2 (8)
C21—C12—C13—C14	1.3 (9)	C28—C27—C32—C23^ii^	180.0 (6)
C11—C12—C13—C14	179.5 (6)	C26—C27—C32—C31	−179.0 (6)
C13—C14—C15—C16	−1.3 (10)	C28—C27—C32—C31	0.7 (8)
C18—C17—C16—C15	173.3 (6)	C29—C30—C31—C32	2.3 (11)
C22—C17—C16—C15	−3.0 (9)	C23^ii^—C32—C31—C30	178.1 (7)
C18—C17—C16—C21	−4.1 (9)	C27—C32—C31—C30	−2.6 (9)
C22—C17—C16—C21	179.5 (5)	C18—O6—C40—C41	−174.7 (5)
C14—C15—C16—C17	−177.9 (6)	C13—O5—C38—C39	−169.5 (7)
C14—C15—C16—C21	−0.3 (9)	C7—O4—C36—C37	163.0 (6)
C18—C17—C22—C23	70.8 (8)	C24—O3—C42—C43	168.7 (9)
C16—C17—C22—C23	−112.8 (7)	C29—O1—C44—C45	177.6 (7)
C13—C12—C21—C20	173.1 (6)	C51—C50—C49—I2	−179.5 (6)
C11—C12—C21—C20	−5.1 (9)	I1—C46—C47—C48	59.3 (10)
C13—C12—C21—C16	−2.9 (9)	C48^ii^—C48—C47—C46	57.8 (9)

Symmetry codes: (i) −*x*+3/2, −*y*+3/2, −*z*+1; (ii) −*x*+1, *y*, −*z*+1/2.
